# The BMIgap tool to quantify transdiagnostic brain signatures of current and future weight

**DOI:** 10.1038/s44220-025-00522-3

**Published:** 2025-10-20

**Authors:** Adyasha Khuntia, David Popovic, Elif Sarisik, Madalina O. Buciuman, Mads L. Pedersen, Lars T. Westlye, Ole A. Andreassen, Andreas Meyer-Lindenberg, Joseph Kambeitz, Raimo K. R. Salokangas, Jarmo Hietala, Alessandro Bertolino, Stefan Borgwardt, Paolo Brambilla, Rachel Upthegrove, Stephen J. Wood, Rebekka Lencer, Eva Meisenzahl, Peter Falkai, Emanuel Schwarz, Ariane Wiegand, Nikolaos Koutsouleris

**Affiliations:** 1 Department of Psychiatry and Psychotherapy, Ludwig-Maximilian-University, Munich, Germany; 2 International Max Planck Research, School for Translational Psychiatry (IMPRS-TP), Munich, Germany; 3 Max-Planck Institute of Psychiatry, Munich, Germany; 4 Department of Psychology, University of Oslo, Oslo, Norway; 5 Section for Precision Psychiatry, Division of Mental Health and Addiction, Oslo University Hospital & Institute of Clinical Medicine, University of Oslo, Oslo, Norway; 6 K.G. Jebsen Center for Neurodevelopmental Disorders, University of Oslo, Oslo, Norway; 7 Department of Psychiatry and Psychotherapy, Central Institute of Mental Health, Medical Faculty, Mannheim, Heidelberg University, Mannheim, Germany; 8 DZPG (German Centre for Mental Health), Partner site Mannheim/Ulm/Heidelberg, Heidelberg, Germany; 9 Department of Psychiatry and Psychotherapy, University of Cologne, Cologne, Germany; 10 Department of Psychiatry, University of Turku, Turku, Finland; 11 Department of Basic Medical Science, Neuroscience and Sense Organs, University of Bari Aldo Moro, Bari, Italy; 12 Department of Psychiatry and Psychotherapy, University of Lübeck, Lübeck, Germany; 13 Department of Psychiatry (Psychiatric University Hospital, UPK), University of Basel, Basel, Switzerland; 14 Department of Neurosciences and Mental Health, Fondazione IRCCS Ca’ Granda Ospedale Maggiore Policlinico, Milan, Italy; 15 Department of Pathophysiology and Transplantation, University of Milan, Milan, Italy; 16 Institute of Mental Health, University of Birmingham, Birmingham, UK; 17 Early Intervention Service, Birmingham Women’s and Children’s NHS Foundation Trust, Birmingham, UK; 18 Department of Psychiatry, University of Oxford, Oxford, UK; 19 NIHR Oxford Health Biomedical Research Centre, University of Oxford, Oxford, UK; 20 School of Psychology, University of Birmingham, Birmingham, UK; 21 Centre for Youth Mental Health, University of Melbourne, Melbourne, Victoria Australia; 22 Orygen, Melbourne, Victoria Australia; 23 Institute for Translational Psychiatry, University Muenster, Muenster, Germany; 24 Department of Psychiatry and Psychotherapy, Medical Faculty, Heinrich-Heine University, Düsseldorf, Germany; 25 DZPG (German Centre for Mental Health), Partner site Munich/Augsburg, Munich, Germany; 26 Hector Institute for Artificial Intelligence in Psychiatry, Central Institute of Mental Health, Medical Faculty, Mannheim, Heidelberg University, Mannheim, Germany; 27 Institute of Psychiatry, Psychology and Neuroscience, King’s College London, London, UK

**Keywords:** Outcomes research, Psychosis, Depression, Schizophrenia, Predictive markers

## Abstract

Understanding the neurobiological underpinnings of weight gain could reduce excess mortality and improve long-term trajectories of psychiatric disorders. Using brain scans from healthy individuals (*n* = 1,504), we trained a model to predict body mass index (BMI) and applied it to individuals with schizophrenia (*n* = 146), clinical high-risk states for psychosis (*n* = 213) and recent-onset depression (ROD, *n* = 200). We computed BMIgap (BMI_predicted_ − BMI_measured_), interrogated its brain-level overlaps with schizophrenia and explored whether BMIgap predicted weight gain at the 1-year and 2-year follow-ups. Schizophrenia (BMIgap = 1.05 kg m^−^^2^) and clinical high-risk individuals (BMIgap = 0.51 kg m^−^^2^) showed increased BMIgap and individuals with ROD (BMIgap = −0.82 kg m^−^^2^) showed decreased BMIgap. Shared brain patterns of BMI and schizophrenia were linked to illness duration, disease onset and hospitalization frequency. Higher BMIgap predicted future weight gain, particularly in younger individuals with ROD, and at 2-year follow-up. Here we show that BMIgap can serve as a potential brain-derived measure to stratify at-risk individuals and deliver tailored interventions for better metabolic risk control.

## Abstract (web summary)

This research investigates the neurobiological factors influencing weight gain, using brain scans from diverse cohorts to develop a predictive model. The findings indicate that BMIgap correlates with psychiatric conditions, suggesting its potential for identifying at-risk individuals and guiding personalized interventions.

## Main

Globally, 26% of adults are overweight (body mass index (BMI) = 25–30 kg m^−^^2^) with an additional 13% classified as obese (BMI ≥ 30 kg m^−^^2^)^1^, highlighting the pandemic nature of obesity^2,3^. Given its strong associations with metabolic diseases, including cardiovascular disease and type 2 diabetes, obesity stands as a major risk factor for somatic disorders^4^. Importantly, obesity frequently parallels psychiatric disorders such as schizophrenia, major depressive disorder, bipolar disorder and personality and anxiety disorders^5,6^. Psychiatric patients have a twofold to threefold higher incidence of obesity and metabolic diseases compared to the general population, substantially contributing to their excess mortality^7,8^. Secondary factors such as smoking, alcohol use, sedentary lifestyle and commonly prescribed medications also markedly affect body weight and metabolic syndromes in psychiatric patients^9,10^. In particular, atypical antipsychotics and antidepressants have been implicated in weight gain^11,12^. Furthermore, individuals exhibiting negative symptoms, which are characterized by affective flattening, anhedonia and avolition, are at an increased risk for weight gain^13^. In turn, weight gain and obesity can have a negative impact on the quality of life and self-esteem of affected individuals, thereby further exacerbating already preexisting mental health issues and ultimately leading to a vicious circle between psychiatric symptoms, secondary disease effects and weight gain^14,15^.

In healthy populations, neuroimaging studies have identified associations between higher BMI and reduced gray matter volume (GMV) in the prefrontal, temporal, parietal and occipital cortices, the cerebellum, insula, thalamus and amygdala^16–18^. These group-level associations might be related to lowered inhibitory control of food-related stimuli resulting from a dysbalance of brain activity in neural systems responsible for cognitive control and reward^17–19^. These findings suggest a plausible relationship between cognitive control deficits and overeating^19^, potentially resulting in higher caloric intake and obesity^17^. Furthermore, in patients with schizophrenia, higher BMI was associated with reduced GMV in the prefrontal cortex, specifically the orbitofrontal cortex, and the hippocampus^20^. In patients with major depressive disorder, higher BMI was correlated with reduced GMV in the medial prefrontal regions, particularly in areas involved in impulse control and emotion regulation^21^. These brain regions are crucial for impulse control and reward processing, suggesting a shared neural basis underlying both psychiatric symptomatology and metabolic dysregulation^22–24^. Furthermore, individuals with schizophrenia exhibit the highest risk for developing metabolic comorbidities among patients with psychiatric disorders. These comorbidities reduce life expectancy up to 25 years in these patients compared to the general population, effects that cannot be explained by medication effects alone^25,26^.

Despite these advances, most existing studies have relied on group-level comparisons, which do not detect individualized deviations from the normative brain–BMI relationship. However, an individualized approach is essential to move beyond population averages and to identify person-specific complex neural markers of metabolic risk. Addressing this gap is especially important in schizophrenia, where metabolic vulnerability may arise from neurobiological alterations that are common to both metabolic and psychiatric disorders. Investigating whether the GMV patterns that predict BMI also contribute to schizophrenia-specific neural changes will provide a framework to examine shared neurobiological mechanisms and quantify brain-based metabolic vulnerability^27,28^. Developing these tools could help detect early alterations in brain–BMI interactions and facilitate targeted interventions, such as exercise, psychotherapy, medications or brain stimulation, to prevent weight gain, improve treatment adherence and reduce excess mortality^29,30^.

To address the knowledge gap in how brain structure relates to BMI in psychiatric disorders, we implemented a normative modeling framework to predict BMI at the individual level using whole-brain GMV trained on a large discovery sample of healthy control (HC) individuals (HC_discovery_). We validated this BMI predictor model in two independent HC samples (HC_validation_ and HC_Cam-CAN_) to measure its generalizability to new, unseen individuals. We then applied the model to clinical groups, including individuals with schizophrenia, recent-onset depression (ROD) and clinical high-risk (CHR) states for psychosis, to examine how brain-based BMI predictions deviate from the reference. The resulting metric, BMIgap (BMI_predicted_ − BMI_measured_), captures individualized brain-based deviations that may reflect metabolic vulnerability toward higher or lower BMI. By comparing BMIgap across clinical groups, we explored whether psychiatric disorders are characterized by systematic deviations in brain–BMI associations, offering new insights into the neurobiological differences underlying metabolic alterations in different psychiatric disorders. Next, to investigate whether BMIgap captures structural brain deviations relevant to schizophrenia and its clinical expression, we examined the phenotypic association between BMIgap, schizophrenia expression (that is, neuroanatomical similarity to schizophrenia) and clinical features using sparse partial least squares (SPLS). Furthermore, to assess the clinical relevance of BMIgap in metabolic outcomes, we correlated it with future weight changes at the 1-year and 2-year follow-ups at the group level and incorporated it alongside additional clinical factors to predict individual weight gain trajectories. We hypothesized that (1) accurate models for individualized BMI prediction can be derived from structural brain imaging using supervised machine learning, (2) interactions between BMI-predictive and disease-specific brain signatures result in systematic brain deviations that are captured by BMIgap, (3) BMIgap is associated with key measures of disease severity, including age at onset, illness duration or hospitalization frequency, and (4) BMIgap can serve as a personalized brain-based tool to assess future weight gain and identify at-risk individuals in the early disease stages.

## Results

### Individualized BMI prediction

**Fig. 1:**
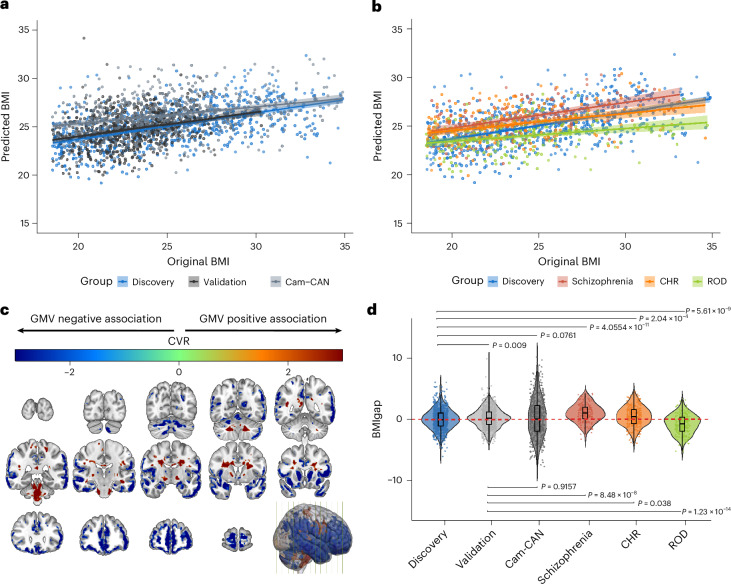
Visualization and performance of the BMI predictor. **a**, Original BMI score versus predicted BMI score with a linear curve fit; the regression line is shown with the ±95% confidence interval (CI) of the mean BMI prediction for the discovery group (blue), validation group (black) and Cam-CAN group (gray). **b**, Original BMI score versus predicted BMI score with a linear curve fit; the regression line with ±95% CI of the mean BMI prediction for the discovery group (blue), patients with schizophrenia (dark orange), CHR individuals (light orange) and individuals with ROD (yellow). **c**, Reliability of the predictive voxels visualized using a grand mean cross-validation ratio (CVR) map thresholded based on the false discovery rate (FDR)-corrected, sign-based consistency map at *α* = 0.05. Cool colors indicate voxels with a negative association of GMV and estimated BMI; warm colors represent a positive correlation. **d**, Box plots of BMIgap for the different study groups, including discovery (*n* = 770), validation (*n* = 734), Cam-CAN (*n* = 536), schizophrenia (*n* = 146), CHR (*n* = 213) and ROD (*n* = 200). The box plots show the median (center line), interquartile range (IQR) (box edges) and whiskers extending to 1.5 times the IQR. BMIgap differences among groups were tested using a two-sided analysis of variance (*F* = 15.97, d.f. = 5, 2,593, *P* = 1.59 × 10^−^^15^).

Sociodemographic characteristics of the discovery, validation, Cam-CAN and patient groups are summarized in Table 1. The model predicted BMI in HC_discovery_ individuals with a mean absolute error (MAE) of 2.75 kg m^−^^2^ (*R*^2^ = 0.28, *P* < 0.001) and generalized to the HC_validation_ with an MAE of 2.29 kg m^−^^2^ (*R*^2^ = 0.26, *P* < 0.001) as well as HC_Cam-CAN_ with an MAE of 2.96 kg m^−^^2^ (*R*^2^ = 0.32, *P* < 0.001) (Fig. 1a). Applied to the clinical subpopulations, the BMI predictor yielded an MAE of 2.85 kg m^−^^2^ for schizophrenia (*R*^2^ = 0.25, *P* < 0.001), an MAE of 3.07 kg m^−^^2^ for CHR (*R*^2^ = 0.16, *P* < 0.001) and an MAE of 2.73 kg m^−^^2^ for ROD (*R*^2^ = 0.10, *P* < 0.001) individuals (Table 2 and Fig. 1b).

**Table 1:** Sociodemographic differences in the discovery, validation, Cam-CAN and patient groups

		HC		*F*/Chi-squared	*P*		Clinical group		*F*/Chi-squared	*P*
	Discovery	Validation	Cam-CAN			Schizophrenia	CHR	ROD		
Sample (*n*)	770	734	536	46.69^a^	**7.253** × **10**^**−11**^	146	213	200	776.60^a^	**2.2** × **10**^**−16**^
BMI, mean (s.d.)	25.10 (4.03)	23.03 (2.07)	25.43 (3.64)	104.8^b^	**4.7637** × **10**^**−44**^	24.02 (3.40)	23.46 (3.42)	24.01 (3.57)	13.48^b^	**1.1456** × **10**^**−8**^
Age, mean (s.d.)	41.26 (15.51)	32.24 (12.75)	54.26 (18.56)	313.44^b^	**2.0873** × **10**^**−119**^	30.83 (9.97)	23.92 (5.24)	26.02 (6.37)	164.42^b^	**3.2518** × **10**^**−86**^
Sex, female, *n* (%)	435 (56.49)	373 (50.82)	261 (48.69)	43.65^a^	**3.32** × **10**^**−10**^	34.00 (23.29)	103 (48.36)	96 (48.00)	590.72^a^	**2.2** × **10**^**−16**^
Symptoms, mean (s.d.)										
PANSS (total)	NA	NA	NA	NA	NA	52.26 (29.45)	52.07 (18.22)	48.44 (14.26)	2.08^b^	0.13
PANSS (positive)	NA	NA	NA	NA	NA	11.92 (8.13)	11.31 (4.16)	8.20 (2.21)	29.09^b^	**9.6792** × **10**^**−13**^
PANSS (negative)	NA	NA	NA	NA	NA	14.98 (9.73)	13.09 (6.86)	12.50 (5.66)	5^b^	**0.007**
PANSS (general)	NA	NA	NA	NA	NA	25.36 (16.13)	27.66 (10.05)	27.74 (8.84)	2.19^b^	0.113
SANS (total)	NA	NA	NA	NA	NA	44.44 (26.52)	27.08 (24.88)	23.91 (20.95)	30.69^b^	**2.4128** × **10**^**−13**^
BDI	3.55 (5.05)	3.27 (5.20)	NA	0.50^c^	0.616	NA	24.09 (11.75)	24.56 (12.48)	182.47^b^	**3.5659** × **10**^**−60**^
Functioning, mean (s.d.)										
GAF:S past month	86.30 (6.43)	87.32 (6.16)	NA	1.54^c^	0.124	NA	51.31 (11.29)	53.92 (13.17)	503.97^b^	**3.0021** × **10**^**−125**^
GAF:D past month	85.49 (6.32)	86.51 (5.89)	NA	1.59^c^	0.113	NA	51.94 (12.)	54.14 (3.60)	385.24^b^	**1.8717** × **10**^**−105**^
GF:S current	8.50 (0.89)	8.54 (0.71)	NA	0.51^c^	0.612	NA	6.25 (1.75)	6.39 (1.23)	157.82^b^	**6.7860** × **10**^**−55**^
GF:R current	8.49 (0.77)	8.59 (0.67)	NA	1.40^c^	0.164	NA	5.80 (1.70)	5.97 (1.81)	150.54^b^	**7.1906** × **10**^**−53**^
WHO QoL (total)	97.45 (33.60)	102.81 (28.91)	NA	1.65^c^	0.099	NA	71.20 (28.81)	74.92 (24.86)	39.58^b^	**8.5257** × **10**^**−17**^
Medications										
Antipsychotics, *n* (%)	NA	NA	NA	NA	NA	NA	60 (28.17)	27 (13.50)	12.51^a^	**4.032** × **10**^**−4**^
Antidepressants, *n* (%)	NA	NA	NA	NA	NA	NA	105 (49.30)	137 (68.50)	4.23^a^	**0.040**
CPZ-equivalent (mg)	NA	NA	NA	NA	NA	358.9 (382.4)	NA	NA	NA	NA

^a^Chi-squared test.

^b^ *F* test.

^c^ *t*-test.

*P* values in bold indicate statistical significance.

BDI, Beck Depression Inventory; GAF:D/I, Global Assessment of Functioning Disability/Impairment Scale; GAF:S, Global Assessment of Functioning Social Scale; GF:R: Global Functioning Role Scale; GF:S, Global Functioning Social Scale; NA, not available; PANSS: positive and negative symptom scale, WHO QoL: World Health Organization quality of life.

**Table 2:** Model performance of the regression analysis for the discovery model and its application to the validation, Cam-CAN and patient groups

Study group	*n*	BMIgap uncorrected (kg m^−^^2^)	BMIgap (kg m^−^^2^)	MAE (kg m^−^^2^)	*R*^2^	*r*	*P*
HC_discovery_	770	−0.01 (3.4)	0 (1.78)	2.75	0.28	0.53	**2.1975** × **10**^**−56**^
HC_validation_	734	1.73 (2.2)	0.23 (1.68)	2.29	0.26	0.51	**1.3696** × **10**^**−15**^
HC_Cam-CAN_	536	0.82 (0.56)	0.25 (3.26)	2.96	0.10	0.32	**7.7240** × **10**^**−14**^
Schizophrenia	146	1.83 (3.0)	1.05 (1.53)	2.85	0.25	0.50	**1.2242** × **10**^**−10**^
CHR	213	1.70 (3.26)	0.51 (1.68)	3.07	0.16	0.40	**2.2209** × **10**^**−7**^
ROD	200	−0.03 (3.48)	−0.82 (1.64)	2.73	0.10	0.32	**3.7118** × **10**^**−5**^

*P* values in bold indicate statistical significance (*α* = 0.05).

BMIgap is shown as the mean ± s.d. (uncorrected and corrected for BMI). Model performance metrics include the MAE, the coefficient of determination (*R*^2^) and Pearson correlation coefficients (*r*, two-sided) between predicted and observed BMI.

Lower GMV in the cerebellar, prefrontal (including the ventromedial prefrontal cortex), occipital and insular cortices, as well as the postcentral gyrus, hippocampus, thalamus, putamen and pallidum (core components of the ventral striatum) and cingulate cortex (involved in reward anticipation, valuation and inhibitory control), was predictive of higher BMI. Lower GMV in the left hemisphere involving the cingulate, cerebellar, inferior occipital and temporal cortices, as well as in the right hemisphere covering parts of the precuneus, putamen and Rolandic operculum were predictive of lower BMI (Fig. 1c, Supplementary Fig. 5 and Supplementary Table 3).

### BMIgap estimation across clinical groups

The application of the BMI predictor yielded a mean (±s.d.) BMIgap of 0.23 (±1.68) kg m^−^^2^ for HC_validation_, 0.24 (±3.26) kg m^−^^2^ for HC_Cam-CAN_, 1.05 (±1.53) kg m^−^^2^ for schizophrenia, −0.82 (±1.64) kg m^−^^2^ for ROD and 0.51 (±1.68) kg m^−^^2^ for CHR individuals. BMIgap differed between HC_discovery_ and HC_validation_ individuals and clinical groups (Fig. 1d; *F*_(discovery versus patients)_ = 33.90, *P* < 0.001; *F*_(validation patients)_ = 32.36, *P* < 0.001). Notably, post hoc pairwise comparisons revealed significant differences in BMIgap between the HC_discovery_ and HC_validation_ groups (*t* = 2.62, *P* = 0.009) probably because of variations in BMI distributions, as indicated by a significant difference in variances (*F* = 3.8018, *P* < 0.001; Supplementary Fig. 1a,c). However, BMIgap did not differ between HC_discovery_ and HC_Cam-CAN_ (*t* = 1.78, *P* = 0.08) as well as HC_validation_ and HC_Cam-CAN_ (*t* = −0.11, *P* = 0.92) (Fig. 1d). BMIgap differed significantly between HC_discovery_ and all clinical groups, with the highest BMIgap for schizophrenia (*t* = 6.68, *P* < 0.001), followed by CHR (*t* = 3.72, *P* < 0.001) and the lowest for ROD (*t* = −5.88, *P* < 0.001).

We did not find significant differences in BMIgap between medication-naive (*n* = 80, 37.56%), antipsychotic-naive (*n* = 153, 71.83%), antidepressant-naive (*n* = 108, 50.70%) and concurrently antidepressant-antipsychotic-treated (*n* = 133, 62.44%) within CHR individuals (*F* = 0.6, *P* = 0.6244). Similarly, no significant differences were observed between the BMIgap of medication-naive (*n* = 59, 29.50%), antipsychotic-naive (*n* = 173, 86.50%), antidepressant-naive (*n* = 63, 31.50%) and concurrently antidepressant-antipsychotic-treated (*n* = 141, 70.50%) individuals with ROD (*F* = 0.002, *P* = 0.9964). Furthermore, BMIgap did not significantly differ between individuals receiving weight gain medications (*n*_CHR_ = 81 (47.65%), *n*_ROD_ = 92 (52.27%), *n*_CHR + ROD_ = 173 (50%)) and those on weight-neutral or no medications (*n*_CHR_ = 28 (16.47%), *n*_ROD_ = 20 (11.36%), *n*_CHR+ROD_ = 48 (13.87%)) within the CHR (*t* = 0.6068, *P* = 0.5448) and ROD (*t* = 1.2187, *P* = 0.2246) groups, nor in the combined CHR and ROD sample (*t* = 0.8445, *P* = 0.3990) (Supplementary Fig. 10). Moreover, we did not find a significant correlation between BMIgap and chlorpromazine equivalents (*r* = −0.01, *P* = 0.86) in the schizophrenia sample.

### Schizophrenia-specific brain signatures

The schizophrenia classifier yielded a balanced accuracy (BAC) of 72.4% (sensitivity = 72.2%, specificity = 72.6%; *P* < 0.001) in separating HCs from individuals with schizophrenia. Voxels predictive of schizophrenia were found predominantly in the inferior, middle and superior frontal gyrus, as well as in hippocampal, thalamic, insular, Rolandic operculum, postcentral, cerebellar and basal ganglia structures. In the right hemisphere, the lingual, fusiform gyrus and mid-temporal lobe were predictive of schizophrenia class membership (Supplementary Fig. 6). The brain patterns of the BMI predictor and the schizophrenia classifier overlapped in the inferior, middle and superior frontal gyrus, caudate, putamen, Rolandic operculum, right precuneus and the middle temporal lobe regions (Supplementary Fig. 7).

### Clinical associations of shared schizophrenia and BMI signatures

**Fig. 2:**
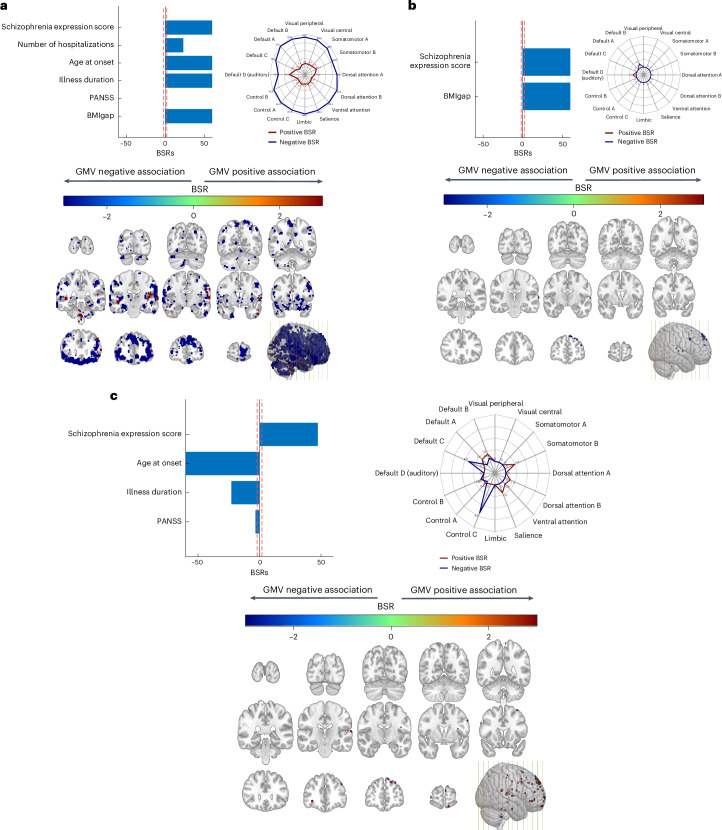
Signatures exhibiting a positive association between schizophrenia expression score and BMIgap effects. **a**, LV2. **b**, LV3. **c**, BMIgap-independent signature of LV5. The bar plots visualize the correlation of each variable with the LV; blue represents variables strongly contributing to the LV. The *x* axis denotes the bootstrap ratios (BSRs) on the *x* axis (interpretable as *z*-scores); the *y* axis denotes the BMIgap, schizophrenia expression score and other clinical items. The red dashed line represents a BSR of 1.96 (equivalent to a 95% CI). The contribution of individual voxels is shown using the BSR in the Montreal Neurological Institute space. Cool colors indicate voxels with a negative correlation of GMV and clinical items; warm colors represent a positive correlation. The spider plots represent the voxel contribution within the 17-network parcellation solution of the Yeo–Buckner atlas^65^. The network names and the cerebral cortical regions that compose the 17 networks are from the supplementary video in ref. ^66^.

The SPLS analysis yielded five reliable latent variables (LVs), representing distinct levels of association between the neuroanatomic overlap regions of the BMI and schizophrenia models, and the clinical disease features (Fig. 2 and Supplementary Fig. 8). While LV2, LV3 and LV5 captured disease-specific patterns, LV1 and LV4 extracted covariate patterns of age and sex (Supplementary Results).

In LV2 (*r* = 0.84, *P* < 0.001), higher BMIgap, schizophrenia expression scores, age at onset, number of hospitalizations and illness duration were related to decreased GMV in the default mode network (DMN) specifically in the A, B and C subcomponents, visual, somatomotor A, attention, salience, limbic, and control networks, and increased GMV in the DMN (auditory) and somatomotor B networks (Fig. 2a).

In LV3 (*r* = 0.85, *P* < 0.001), higher BMIgap and higher schizophrenia expression scores were related to decreased GMV in the DMN-B and increased GMV in the DMN D (auditory) (Fig. 2b).

In LV5 (*r* = 0.58, *P* < 0.001), lower PANSS total score, illness duration and age at onset, and higher schizophrenia expression score were related to decreased GMV in DMN-C and control-C networks, and increased GMV in the DMN-A/B, somatomotor B, dorsal attention-B and salience networks (Fig. 2c).

### BMIgap and future weight change

**Fig. 3:**
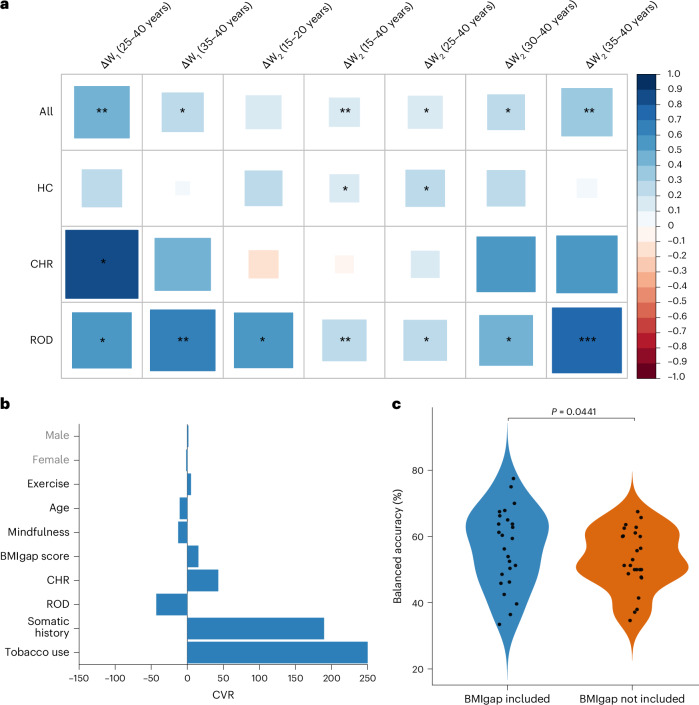
Association between BMIgap and weight change. **a**, Heatmap showing the correlations between BMIgap and weight change (Δ*W*_1_ = weight_T1_ − weight_T0_; Δ*W*_2_ = weight_T2_ − weight_T0_) across different age ranges and clinical groups (HC, CHR, ROD). Correlation coefficients were calculated using two-sided Pearson correlations. Significance was assessed with FDR correction for multiple comparisons; significant values are denoted as *P ≤ 0.05, **P ≤ 0.01 and ***P ≤ 0.001, with exact (FDR-adjusted) *P* values reported in Supplementary Tables 7–10. The plot uses blue squares to represent positive correlations and red squares for negative correlations. The size of each square corresponds to the magnitude of the correlation value, with larger squares indicating stronger correlations. **b**, Feature importance analysis for predicting 7% weight gain, expressed as the CVR. **c**, Comparison of balanced accuracy for predicting 7% weight gain at T2, with and without BMIgap as a feature. Model performance was compared using a two-sided paired *t*-test (*t* = 1.907, d.f. = 24, *P* = 0.0441).

In HC individuals, BMIgap showed a positive correlation with a 2-year weight gain (Δ*W*_2_, *n* = 216; *r* = 0.14, *P*_FDR_ = 0.0347); specifically, in the subgroup of adults between 25 and 40 years (N = 46; *r* = 0.46, *P*_FDR_ = 0.034). The BMIgap of individuals with ROD positively correlated with both Δ*W*_1_ (*n* = 141; *r* = 0.18, *P*_FDR_ = 0.053) and Δ*W*_2_ (*n* = 92; *r* = 0.30, *P*_FDR_ = 0.05) at all ages and notably for young individuals aged between 15 and 20 years (Δ*W*_2_: *n* = 15; *r* = 0.52, *P*_FDR_ = 0.051). Moreover, females with ROD showed a significant correlation between BMIgap and weight gain at T1 (Δ*W*_1_: *n* = 70; *r* = 0.29, *P* = 0.04), while males did not (*n* = 71; *r* = 0.02, *P* = 0.86). CHR individuals between 25 and 40 years of age showed significant correlations between BMIgap and Δ*W*_1_ in the +5% weight increase subgroup (*n* = 58; *r* = 0.29, *P*_FDR_ = 0.047) and with Δ*W*_2_ specifically in the +3% (*n* = 39; *r* = 0.28, *P*_FDR_ = 0.049), +5% (*n* = 32; *r* = 0.31, *P*_FDR_ = 0.049) and +7% (*n* = 21; *r* = 0.27, *P*_FDR_ = 0.01) subgroups (Fig. 3a, Supplementary Fig. 9 and Supplementary Tables 7–10).

### Weight gain predictor

The multivariate weight gain prediction model indicated a weight gain of +7% at T2 with a BAC of 59.2% (sensitivity = 64.9%, specificity = 53.5%, *P* < 0.05); the +3% (BAC = 52.2%; sensitivity = 52.5%, specificity = 51.9%, *P* = 0.21) and +5% weight gain prediction models did not perform above chance level (BAC = 45.2%, sensitivity = 62.5%, specificity = 35.2%) (Supplementary Table 4). Key predictive features for the 7% weight gain predictor included age, clinical group (ROD, CHR), exercise, somatic history, BMIgap and tobacco use. Specifically, among CHR individuals, a higher BMIgap, combined with a history of somatic conditions and reduced mindfulness-based exercises was predictive of weight gain. For individuals with ROD, BMIgap was negatively associated with 7% weight gain, particularly in individuals with a history of lower somatic comorbidities, reduced tobacco use and older age (Fig. 3c). Notably, the predictive performance of the +7% weight reduced after excluding BMIgap as a feature (BAC = 56.1%; sensitivity = 48.6%, specificity = 63.6%; *P* < 0.05); these two models differed significantly (*P* < 0.05) (Fig. 3c).

## Discussion

The aim of our study was to introduce BMIgap as a metric to evaluate BMI-related brain signatures, interrogate its overlaps with schizophrenia brain patterns and explore its implications for future weight gain.

The BMI predictor relied on lower GMV in the prefrontal and temporal regions, particularly in areas associated with reward and inhibitory control and higher GMV in the precuneus, putamen and Rolandic operculum to predict BMI. When comparing BMIgap across samples, we found no notable difference between the HC_discovery_ and the independent HC_Cam-CAN_ cohorts, suggesting that our model generalized well to external healthy populations. This was further supported by a notable correlation between BMI_predicted_ and BMI_observed_ in HC_Cam-CAN_. Moreover, individuals in the psychosis spectrum (schizophrenia and CHR) showed an increased BMIgap while individuals with affective diseases (ROD) showed a decreased BMIgap. Moreover, we found that the separability of schizophrenia from HCs partly overlapped with BMI-related structural brain variation pertaining to the inhibitory control and reward systems. SPLS analysis revealed that a prefrontal-temporal brain pattern predicting both disease and BMI phenotypes was associated with longer illness, later disease onset and higher number of hospitalizations. At a group level, higher BMIgap was correlated with future weight gain, an effect particularly pronounced in the longer-term trajectories of younger individuals with depressive disorders. At an individual level, BMIgap was identified as a predictive feature for future weight gain in combination with age, clinical group (ROD and CHR), exercise, history of somatic comorbidities and tobacco use.

The co-occurrence of higher BMI and lower GMV in the reward and salience systems in the BMI predictor model may represent a neurobiological mediator of eating behaviors^16–19^. Furthermore, BMI-predictive GMV reductions in brain areas related to taste, reward and inhibitory control, may contribute to increased susceptibility to hypercaloric eating^17,19^. These GMV alterations align with dysfunctional reward anticipation and impaired inhibitory control, which have been implicated in heightened food cravings, compulsive eating patterns and reduced sensitivity to homeostatic satiety signals^17,19^. Additionally, given the overlap between the BMI-predictive and schizophrenia signatures within inhibitory, reward and cognitive control regions, this suggests an underlying neural vulnerability between obesity and schizophrenia that is consistent with previous research^20,23^. Our findings substantiate previous reports that prefrontal deficits might lead to reduced cognitive control and may therefore amplify the risk of addictive behaviors, such as overeating, which could substantially contribute to an increased BMI in psychiatric patients^17,18,23^. We did not observe notable differences between HC_discovery_ and HC_Cam-CAN;_ BMI_predicted_ and BMI_observed_ for HC_Cam-CAN_ strongly correlated, suggesting that our model generalized well across independent HC samples. When applied to clinical groups, we observed a positive BMIgap for the schizophrenia and CHR groups and a negative BMIgap for the group with ROD compared with the HC_discovery_. The positive BMIgap in schizophrenia and CHR suggests that these groups exhibit brain structural patterns typically associated with HCs who have a higher BMI, despite having lower actual BMI than predicted. This may reflect underlying pathophysiological processes, such as neuroinflammation, insulin resistance or gut–brain axis disruptions, which are known to affect both metabolic regulation and brain structure^31^. In schizophrenia, these effects may be more pronounced than in CHR because of longer illness duration and associated metabolic burden, potentially predisposing individuals to future weight gain. Conversely, the group with ROD group a negative BMIgap, indicating that their brain structural patterns more closely resemble those of HCs with a lower BMI, despite having higher actual BMI than predicted. This finding may relate to early-stage depressive phenotypes, often characterized by appetite suppression and reduced energy intake, and may reflect a distinct neurobiological profile in depression compared to psychotic disorders. These interpretations align with prior evidence linking early or untreated depression to reduced appetite, weight loss, altered energy metabolism, and abnormal hypothalamic–pituitary–adrenal signaling in patients with comorbid depression and anorexia nervosa^32–34^. The significance of BMIgap lies in its ability to capture systematic brain-based alterations in BMI prediction across different psychiatric populations, highlighting distinct patterns of brain–metabolism interactions that may not be fully explained by traditional anthropometric measures such as BMI.

Importantly, our analyses suggest that BMIgap is largely unaffected by the intake of weight-gain versus weight-neutral medication, indicating that brain-based BMI deviations may reflect more stable neurobiological traits rather than being predominantly driven by pharmacological effects. This further supports the utility of BMIgap as a potential tool of brain–metabolic alterations, independent of current medication status. Nonetheless, future studies with larger samples and longitudinal designs are needed to explore potential medication-related changes in brain-based BMI predictions over time.

Moreover, occurrence of metabolic syndromes in psychiatric patients is often linked to psychiatric medication, namely antipsychotics and antidepressants^11,35,36^. Antipsychotics affect the mesolimbic dopaminergic system and the ventromedial nucleus, thereby altering behavioral responses to environmental stimuli and regulating both food intake and body weight^35^. Antidepressants affect metabolic risk by increasing appetite and suppressing satiety, primarily through histaminergic and serotonin receptor antagonism^11,12^. However, we did not find notable differences when comparing the BMIgap of fully naive, partly naive and concurrently antidepressant-antipsychotic-treated CHR or ROD. Moreover, we did not observe any clear associations between BMIgap and antipsychotic dosage in patients with schizophrenia. These findings indicate that BMIgap captures a neurobiological BMI signature, which may represent a more disease-specific or individual predisposition toward future weight changes. This finding aligns with previous research indicating that patients with psychiatric disorders are at an increased risk of developing metabolic syndrome and obesity, independent of medication use^37,38^, contrasting other literature that primarily attributes weight gain to the effects of psychiatric medication^11,12^.

The phenotypic association analysis linked control and reward brain networks to schizophrenia diagnosis, BMIgap and clinical variables in unique ways. The multivariate signatures of concurrently high BMIgap and schizophrenia expression scores (LV2, LV3) were associated with both a decrease in GMV within the limbic network and increased GMV within the DMN (auditory) network. In obesity research, these networks have been involved in reward processing^39^, food motivation^40^ and executive and affective control^41^; in schizophrenia research, they have been particularly implicated in the impaired processing of negative emotions^42^. Furthermore, later disease onset, higher hospitalization frequency and longer illness, were associated with both higher BMIgap and schizophrenia expression scores, thereby implying a potential association between the severity of schizophrenia and the presence of obesity risk traits. Moreover, we identified a pattern independent of BMIgap (LV5) suggesting that there are distinct effects of schizophrenia and BMI on the brain, indicating that those with early onset, shorter illness and milder symptoms are less likely to show high schizophrenia diagnostic separability, thus highlighting the complexity of schizophrenia subtypes and the variability in its manifestation and progression.

Moreover, our assertion that BMIgap is associated with future weight changes was supported by the positive correlation between BMIgap and future weight change observed across all groups, with the strength of this correlation increasing from the 1-year to the 2-year follow-ups. Notably, these correlations were most pronounced among young individuals (15–20 years) in the group with ROD and young adults (25–40 years) in the CHR group, indicating that individuals in the early stages of psychiatric illness may exhibit distinct brain signatures that predispose them to future weight gain. Furthermore, the 7% weight gain predictor highlighted that young adults with ROD who have higher exercise rates, use less tobacco, have less somatic comorbidities and lower BMIgap are less likely to experience weight gain. Conversely, younger CHR individuals with reduced exercise rates, more history of somatic comorbidities, higher tobacco use and higher BMIgap were at an increased risk for future weight gain. Notably, exclusion of the BMIgap feature from the 7% weight gain predictor led to a marked reduction in predictive accuracy of the model in correctly predicting future weight gain.

The translational value of BMIgap lies in its potential to identify individuals at risk for future weight changes, especially weight gain, in early disease stages, and particularly in vulnerable, at-risk populations such as CHR individuals and individuals with ROD. Assessing BMIgap could potentially enable identification of individuals who, despite having a BMI in the normal range, are in fact, neurobiologically, at increased risk for future weight gain. This could lead to stratified, targeted and timely interventions, such as early access to guided physical exercise or add-on medication such as metformin^43,44^. More specifically, given its association with reward and inhibitory control networks, patients with a high BMIgap may benefit from targeted psychotherapy aimed at enhancing self-regulation, impulse control and reward sensitivity, which are key factors influencing eating behavior and metabolic health^41^. Approaches such as cognitive behavioral therapy and inhibitory control training enhance executive function, decision-making and impulse control in relation to food intake^41,45^. Additionally, dopaminergic modulation may help regulate reward processing and compulsive eating behaviors, particularly in individuals exhibiting heightened reward sensitivity to hypercaloric foods^46^. All of this would eventually be aimed at preventing weight gain and associated metabolic syndromes in at-risk individuals and thus improve treatment adherence, overall quality of life and treatment outcomes^29,30^.

## Limitations

One limitation of our study is the lack of individuals with very high (>35 kg m^−^^2^) or low (<18 kg m^−2^) BMI, which limits the generalizability of our findings to patients with comorbidities related to overweight (for example, type 2 diabetes, hypertension) or underweight (for example, anorexia). The absence of harmonized clinical and longitudinal data across cohorts, particularly the lack of weight follow-up data in the group with schizophrenia, limited our ability to assess BMIgap’s association with future weight changes in this population. More broadly, the lack of standardized clinical and medication data is a common limitation in multisite studies^47–49^. Future research could address these challenges by systematically collecting metabolic, medication-related and clinical factors influencing brain structure and psychiatric conditions. Such efforts should extend to early-stage populations, including CHR and ROD, as well as a broader range of psychiatric disorders, such as posttraumatic stress disorder and obsessive–compulsive disorder. Furthermore, our study did not include metabolic markers such as lipid profiles, fasting glucose or homeostatic model assessment for insulin resistance, nor precise obesity measures like waist-to-hip ratio and body fat percentage. While BMI provided a practical and widely used index for this endeavor, future research incorporating a wider, potentially more comprehensive array of metabolic and anthropometric markers is warranted to deepen our insights into brain–metabolism interactions in psychiatric populations^16–18,50^. Finally, pubertal status was not assessed in the 15–20-year-old subgroup, which may have influenced weight-related trajectories and represents a limitation of the current analysis.

## Conclusion

In conclusion, our study identified BMIgap as a crucial metric for exploring the relationship between BMI and brain structure in psychiatric populations, particularly within the depression and schizophrenia spectrum. We found that elevated BMIgap was associated with pronounced neurobiological alterations in reward and inhibitory control systems, indicating a complex interplay between obesity and schizophrenia. Our findings further suggest that BMIgap could potentially be a predictive indicator of future weight gain, especially among younger individuals with a higher disease burden. Therefore, BMIgap could serve as a template for machine learning and brain imaging to enhance the early identification of patients at risk for metabolic complications. Integrating BMIgap, or future, more sophisticated tools, into clinical assessments may improve strategies for preventing weight gain in psychiatric patients.

## Methods

### Sample

The study followed the Transparent Reporting of a Multivariable Prediction Model for Individual Prognosis or Diagnosis reporting guidelines (www.equator-network.org/reporting-guidelines/tripod-statement/). We included T1-weighted magnetic resonance imaging (MRI) scans of 1,504 HCs (HC_discovery_: *n* = 770, age = 41.3 ± 15.5 years, 56.5% female; HC_validation_: *n* = 734, age = 32.2 ± 12.8 years, 50.8% female; HC_Cam-CAN_: *n* = 536, age = 54.3 ± 18.6 years, 48.7% female) from five independent datasets covering 15 sites: Information eXtraction from Images (IXI) (https://brain-development.org/ixi-dataset/); Personalized Prognostic Tools for Early Psychosis Management (PRONIA) (www.pronia.eu); the Norwegian Centre for Mental Disorders Research (NORMENT) (www.med.uio.no/norment/)^51^; the Munich Brain Imaging Database (MUC^52^); and the Cambridge Centre for Ageing and Neuroscience (Cam-CAN^53,54^) datasets (Supplementary Methods). Moreover, three patient populations were included: individuals with schizophrenia (*n* = 146, age = 30.8 ± 10.0 years, 23.3% female) from the MUC cohort and CHR (*n* = 213, age = 23.9 ± 5.2 years, 48.4% female) and individuals with ROD (*n* = 200, age = 26.0 ± 6.4 years, 48.0% female) from the PRONIA study (Table 1 and Supplementary Table 2). All participants gave informed consent and received compensation. All studies were approved by their local ethics committee and adhered to the ethical standards outlined in the Declaration of Helsinki^55^. All datasets were collected in collaboration with local research teams, who contributed to study design, implementation and authorship, ensuring compliance with local ethical standards and relevance.

### Modeling framework

In our study, we developed an individualized BMI prediction model based on voxel-wise GMV. This strategy builds on previous work by Opel et al.^18^, who applied multivariate models to predict BMI from GMV in HCs^18^. We extended this approach by implementing a normative modeling framework that not only enables individualized predictions but also quantifies individualized deviations from expected, that is, ‘normative’, brain–BMI relationships. This allowed us to derive a brain-based vulnerability measure, BMIgap, which captures how much an individual’s brain structure deviates from typical BMI-related patterns. We then applied this model to clinical populations to investigate brain-based metabolic vulnerability across different psychiatric conditions. A detailed explanation of our modeling framework and sample selection procedure is provided in the (Supplementary Methods).

### Participant selection

We included HCs aged 15–75 years with a BMI range of 18.5–35 kg m^−^^2^ without current or previous psychiatric disorders in the discovery model by following these steps: (1) to avoid an underrepresentation of the tails of the BMI distribution and to maximize model generalizability throughout the investigated BMI range, we selected 770 HCs from a sample of 1,504 HCs and distributed them into 33 BMI bins (0.5 BMI per bin) from the 18.5–20.0 bin to the 34.5–35.0 bin, aiming for an equal number of individuals per bin to reduce bias toward the overrepresented normal BMI range and improving generalizability to individuals with high or low BMI (Supplementary Fig. 1a); (2) to control for the natural correlation between BMI and age, age distribution was matched in each BMI bin wherever possible, so that in addition to equal numbers of individuals across the BMI range, the 33 BMI bins had a comparable mean age^56^ (Supplementary Fig. 1c). These 770 HCs constituted the HC_discovery_ sample; the remaining 734 HCs constituted the HC_validation_ sample, while 536 HC individuals from the Cam-CAN dataset (HC_Cam-CAN_) constituted an additional external validation sample for our model. The clinical population consisted of individuals with schizophrenia (*n* = 146), CHR (*n* = 213) and ROD (*n* = 200) (Supplementary Figs. 1 and 2 and Supplementary Methods).

### MRI data acquisition and preprocessing

Participants in the IXI and PRONIA studies underwent MRI scanning at 1.5T or 3T, while NORMENT, Cam-CAN and MUC participants were examined with 1.5T MRI scanners (Supplementary Methods and Supplementary Table 1). To facilitate between-study comparability, we applied the VBM8 preprocessing pipeline described in ref. ^18^ to produce normalized, modulated GMV tissue maps (Supplementary Methods). For computational efficiency and noise reduction, GMV images were resliced to a 3 × 3 × 3-mm^3^ isotropic voxel resolution.

### Machine learning analysis

The open-source machine learning software NeuroMiner (v1.1) (https://github.com/neurominer-git/NeuroMiner_1.1) was used for the training and application of all supervised machine learning models. To prevent information leakage between training and test data, thereby limiting overfitting risk and enhancing model generalizability, we implemented a repeated nested cross-validation with five folds and five permutations each on the inner and outer cross-validation cycles. All preprocessing parameters were computed using the training data of the inner cross-validation cycle and applied to the data of the inner test and outer validation folds. Voxel-wise GMV data preprocessing included: (1) Gaussian smoothing with 0-mm, 3-mm, 6-mm and 9-mm full-width at half-maximum kernel widths; (2) regressing out age effects using partial correlation analysis; (3) mean offset correction to remove site effects, that is, scanner effects; (4) principal component analysis with different energy levels (0.25, 0.50, 0.75) to reduce the dimensionality of the image space; and (5) voxel-wise scaling from 0 to 1. The models were then trained on the inner training folds and applied to the inner test and outer validation folds, ensuring that validation remained completely independent of training. We used *ν*-support vector machine regression with a linear kernel to predict BMI based on whole-brain voxel-wise GMV (71,276 features) using the MAE as the optimization criterion. The statistical significance of the model was evaluated using 1,000 permutations of the BMI label (significance level *α* = 0.05). Predictive brain patterns were visualized at the voxel level combining the grand means of the CVR^57^ and sign-based consistency mapping, thereby assessing whether a feature consistently predicted higher or lower BMI across cross-validation partitions^58^ (Supplementary Methods). We applied this BMI predictor to the HC_validation_ and HC_Cam-CAN_ cohorts to assess the model’s generalizability. Furthermore, the BMI predictor was applied to individuals with schizophrenia, ROD and CHR to obtain brain-based BMI predictions for these clinical populations.

### BMIgap calculation

BMIgap was calculated by subtracting the measured BMI from the brain-based predicted BMI (BMIgap = BMI_predicted_ − BMI_measured_). A positive BMIgap indicates that an individual’s brain structure resembles that of someone with a higher BMI than their actual BMI measurement, whereas a negative BMIgap suggests the opposite. To assess systematic deviations within groups, we compared BMIgap values across diagnostic categories (HC, schizophrenia, ROD, CHR), allowing us to quantify whether specific populations consistently exhibit overestimations or underestimations in brain-based BMI relative to the reference sample. To mitigate systematic bias in predicted BMI, characterized by overestimation at lower BMI ranges and underestimation at higher BMI ranges, BMIgap was adjusted for BMI using partial correlation analysis^59^ (Supplementary Methods and Supplementary Figs. 3 and 4). We used the corrected BMIgap for all further analysis steps. Furthermore, to investigate potential medication effects on BMIgap, we conducted additional analyses. First, we independently calculated BMIgap for antipsychotic-naive and antidepressant-naive individuals with CHR and ROD to investigate whether medication influenced BMIgap in these subpopulations. Next, we categorized individuals in the CHR and ROD groups based on whether they were receiving weight-gain-associated medications (for example, mirtazapine, olanzapine, clozapine, quetiapine, chlorpromazine) or weight-neutral medications (for example, bupropion, lurasidone, ziprasidone) at baseline and compared BMIgap values across these subgroups^43,44^. As there were no unmedicated patients with schizophrenia, we correlated BMIgap with their chlorpromazine equivalents.

### Clinical investigation of BMIgap

To understand the clinical implications of BMIgap, we analyzed its relationship with clinical variables, particularly within the group with schizophrenia. To identify brain patterns distinguishing individuals with schizophrenia from HCs, we trained a schizophrenia (*n* = 146) versus HC_discovery_ classifier in the MUC sample (*n* = 133), using the same preprocessing and cross-validation settings as the BMI prediction model. This classification allowed us to assess whether BMI-predictive GMV regions overlap with GMV regions distinguishing individuals with schizophrenia from HCs, indicating potential shared neuroanatomical substrates.

The binarized sign-consistency maps derived from the schizophrenia and BMI predictors were overlapped to identify brain regions commonly predictive of both phenotypes (Supplementary Methods). Furthermore, we extracted the decision scores from the schizophrenia classifier, which we refer to as the schizophrenia expression score. A higher schizophrenia expression score indicates a higher likelihood to be classified as having schizophrenia and therefore a greater neuroanatomical similarity to schizophrenia, while a lower schizophrenia expression score reflects higher likelihood of HC classification and neuroanatomical HC similarity.

Next, we studied the covariation between BMIgap, schizophrenia expression and clinical variables within the overlapping brain regions of schizophrenia and BMI. To this end, we used multivariate SPLS using the SPLS Toolbox described in refs. ^60,61^ to investigate the covariance patterns between two data domains in the schizophrenia sample: (1) a six-feature matrix, including BMIgap, schizophrenia expression scores, PANSS total score, age at onset, illness duration and number of hospitalizations; and (2) a brain data matrix containing the vectorized voxels extracted using the binarized mask of overlapping BMI-predictive and schizophrenia-predictive voxels. The SPLS algorithm uses singular value decomposition to generate multiple layers of distinct, multivariate associative effects between the two data matrices, called LVs (Supplementary Methods).

### Investigation of BMIgap and future weight change

We investigated the association between BMIgap and future weight change using two approaches: (1) correlation analysis; and (2) machine learning-based prediction of weight gain. In the PRONIA cohort, where longitudinal data were available, we correlated BMIgap to weight changes at the 1-year (T1) and 2-year (T2) follow-ups. Weight changes were calculated as the difference between the weight at the follow-up time point and the weight at baseline (T0): 1 year (Δ*W*_1_ = weight_T1_ − weight_T0_); 2 years (Δ*W*_2_ = weight_T2 _− weight_T0_). The same age range stratifications were applied consistently across both time points to ensure comparability in our analyses. Correlation analyses were conducted for the entire cohort, and separately for sex and study group. In the first step, we analyzed the correlation between BMIgap and all observed weight changes. Building on the previous literature, we correlated BMIgap to weight changes only within subpopulations of patients who exhibited at least a +3%, +5% or +7% weight gain at the respective follow-up^62–64^. Additionally, to examine potential age-related effects, we analyzed these correlations across several age ranges, including broader spans (15–40, 20–40, 25–40, 30–40 and 35–40 years) and finer-grained 5-year intervals (15–20, 20–25, 25–30, 30–35 and 35–40 years). Finally, we used the three weight gain thresholds (+3%, +5% and +7%) as classification criteria to predict whether individuals with CHR and ROD experienced weight gain above these thresholds at T1 and T2 or not. We used BMIgap as well as age, sex, study group (ROD, CHR), exercise (strenuous exercise or mindfulness activities such as yoga and meditation) and history of somatic comorbidities (that is, whether the individual suffered from somatic illness) as features. Classification analyses were conducted with and without BMIgap as a feature to assess if BMIgap significantly affects the prediction of future weight gain (*P* < 0.05).

### Reporting summary

Further information on research design is available in the Nature Portfolio Reporting Summary linked to this article.

## Supplementary information

Supplementary Information Supplementary Figs. 1–10, Tables 1–10, Methods and Results.

Reporting Summary

## Data Availability

Parts of the data, including the IXI dataset (https://brain-development.org/ixi-dataset/) and the CAM-CAN dataset (https://camcan-archive.mrc-cbu.cam.ac.uk), are accessible to researchers upon request from the respective repositories. Other datasets (PRONIA, MUC, NORMENT) analyzed during the current study are not publicly available because of data sharing restrictions defined in the participants’ signed informed consent agreements. Trained models are available from the corresponding author upon reasonable request.

## Code availability

The code supporting the findings of this study is available via GitHub at https://github.com/adyasha95/BMIgapCodeRepo.
