# Supplementary material for: The BMIgap tool to quantify transdiagnostic brain signatures of current and future weight
Source: Nat Ment Health. 2025 Oct 20;3(11):1395–406. doi: 10.1038/s44220-025-00522-3 (PMC12589130; doi:10.1038/s44220-025-00522-3)
Supplement: Supplementary file 1 — Supplementary Figs. 1–10, Tables 1–10, Methods and Results. [file 44220_2025_522_MOESM1_ESM.pdf]

nature mental health

Supplementary information https://doi.org/10.1038/s44220-025-00522-3

The BMIgap tool to quantify transdiagnostic brain signatures of current and future weight

In the format provided by the authors and unedited

SUPPLEMENTAL MATERIAL

Supplementary Methods

PRONIA cohort

PRONIA (Personalized Prognostic Tools for Early Psychosis Management, https://www.pronia.eu/) is a multisite study where participants were recruited across nine sites in Finland, Germany, Italy, Switzerland, and the United Kingdom following the_standardized recruitment and ascertainment protocol of the study. The observational part of the protocol involved follow-up examinations every three months after the index ascertainment which was further implemented by the nine PRONIA sites. The participants were pseudonymized twice after recruitment locally at each site and centrally in the PRONIA portal. This portal has a multi-user database which hosts the clinical and neurocognitive information, and defaced MR images of the study participants which are organized into digital questionnaires, visits, and cases. The portal also provided the case managers with the control of a web-based interface for entering and uploading the different acquired data into the respective questionnaires. Furthermore, there was an implemented PRONIA@home mobile device interface allowing study participants to securely log into the portal and fill out the self-rating questionnaires of a given visit. The data is checked by an automatic quality control procedure on completion of data entry across all questionnaires of a given visit which executes approximately 1600 data integrity and dependency rules: (i) basic checks on missing data and data ranges, (ii) checks on dependency within one questionnaire, (iii) dependencies between two questionnaires within one visit, (iv) dependencies between two consecutive visits (such as consistency of dates). The errors found are reported back to the case managers, thus allowing for a manual correction of the respective issues. The process is reiterated until the given visit's quality of clinical questionnaires is sufficient for the visit to be locked.

Participant inclusion

In the current study, we included: (i) Information eXtraction from Images (IXI; https://brain-development.org/ixi-dataset/), Personalized Prognostic Tools for Early Psychosis Management (PRONIA; www.pronia.eu), Norwegian Centre for Mental Disorders Research (NORMENT; https://www.med.uio.no/norment/ (1)) and Munich Brain Imaging Database (MUC, (2)) healthy individuals who had received a structural MRI scan and further were successfully processed by the VBM8 pipeline, (ii) healthy subjects which had the information about height, weight, age and sex (participants within 18.5 - 35 kg/m^2 of body mass index (BMI) and 15-75 years of age were included), (iii) Schizophrenia patients which had 70% of the clinical items and complying to (i) and (ii).

The patients with schizophrenia were evaluated with the Structured Clinical Interview for Diagnostic (SCID) and Statistical Manual of Mental Disorders, Fourth Edition, for Axis I Disorders. The criteria for recent onset-depression (ROD) were a first major depressive episode within the past three months, as determined by the Structured Clinical Interview for DSM-IV-TR (SCID) (29). The clinical high-risk states (CHR) for psychosis state were defined by: (i) cognitive disturbances as evaluated with the Schizophrenia Proneness Instrument, and/or (ii) ultra-high-risk criteria for psychosis, as outlined by the Structured Interview for Psychosis-Risk Syndromes. Inclusion criteria for ROD and CHR individuals only allowed minimal antipsychotic medication. Additional general exclusion criteria for PRONIA subjects are described in Table S2 and in Koutsouleris et al. 2018 (3).

MRI preprocessing pipeline

Using the VBM8 toolbox (http://dbm.neuro.uni-jena.de/vbm), the structural images were first bias-corrected, tissue classified and normalized to MNI-space. The normalization used linear (12-parameter affine) and non-linear transformations, within a unified model including high-

dimensional DARTEL-normalization. Next, the grey matter segments were modulated only by the non-linear components to retain the actual grey matter values locally, thereby, there was no need to correct for total intracranial volume. An absolute threshold masking with a threshold value of 0.1 was further used, as recommended in VBM8 analyses.

Creating gray matter volume-specific brain mask

To generate the binarized gray matter volume (GMV) brain mask, we employed the ImCalc function within the Statistical Parametric Mapping (SPM) toolbox. The first image comprised preprocessed data from a single subject, utilizing the desired voxel size. The second image corresponded to the Tissue Probability Mask (TPM) specific to GMV within the SPM toolbox, previously utilized in tissue segmentation. Specifically, for GMV, we retained only voxels with values >0.2 in the second image (TPM). Subsequently, the ImCalc function resliced the TPM GMV-specific mask according to the specifications of our input image, resulting in a binarized mask that retained GMV voxels >0.2 (4).

Modelling framework

Normative modeling is a statistical approach used to learn a reference distribution (i.e., "norm") from a representative sample and assess how new individuals deviate from it. Normative modelling has been widely adopted in neuroscience to capture individual-level deviations from expected brain-behavior relationships. For example, BrainAGE models estimate the difference between predicted brain age and chronological age from structural MRI, offering a biomarker of accelerated or delayed brain aging (5,6). Other studies have applied normative frameworks to model cognitive performance trajectories (7) and clinical symptom dimensions, enabling the identification of individuals who deviate from healthy reference distributions in conditions such as schizophrenia, autism, and depression (8,9). The advantage of normative modeling lies in its ability to provide individualized deviation metrics, which move beyond group

comparisons and offer insights into individual-level alterations. Furthermore, most machine learning studies in psychiatric neuroimaging have focused on training models to differentiate between diagnostic groups, such as patients and healthy controls (10,11). While effective for identifying group-level differences, these classification-based approaches often obscure person-specific patterns of deviation, as they are optimized to detect features that separate groups rather than capture individual variability. Instead, normative modeling helps us understand how much a single individual deviates from a normative, "healthy", population. In our case, it reflects how a person's brain structure differs from what is expected, providing a useful way to capture meaningful individual differences (1,8). In the present study, we adopted a normative modeling strategy that serves a similar purpose: establishing a brain-based reference model of BMI from healthy individuals, against which individual deviations in clinical subpopulations can be quantified.

To construct the reference model, we defined a discovery sample of 770 psychiatrically healthy individuals aged 15–75 years with BMI values between 18.5 and 35 kg/m², recruited across 15 sites. Importantly, this sample was designed to approximate a uniform BMI distribution, rather than relying on a naturally skewed distribution commonly seen in population data. Specifically, individuals were divided into 33 BMI bins (0.5 BMI per bin), and an equal number of participants were selected from each bin. This uniform sampling strategy ensured that the model was trained across the full spectrum of BMI values, with equal amounts of individuals from all 33 bins, thereby reducing bias toward the overrepresented normal-weight range and improving generalizability to individuals with high or low BMI. This approach amounted to a downsampling of the HC population, which also allowed us to better capture the full variability in BMI-related GMV patterns. To control for the natural correlation between BMI and age, the age distribution was matched within each BMI bin, so that in addition to equal numbers of individuals from the entire BMI range, all 33 BMI bins had a comparable mean age.

While these individuals were psychiatrically healthy, they included both normal-weight and overweight individuals. This choice improves the ability of the model to predict BMI across its full range and to serve as a reference for individualized brain-based deviations in clinical populations.

A support vector regression model was then trained using voxel-wise GMV data (71276 features) as predictors and BMI as the label. This model learned distributed GMV patterns that best predict BMI within the HC discovery sample. Once trained, the model was applied to unseen individuals to generate predicted BMI values ($BMI_{predicted}$) based solely on their brain structure.

To quantify how much a person's brain structure deviates from the expected norm, we computed BMIgap as the difference between $BMI_{predicted}$ and actual BMI ($BMI_{measured}$), i.e. BMIgap = $BMI_{predicted}$ - $BMI_{measured}$. A positive BMIgap indicates that an individual's brain resembles that of someone with higher BMI than their actual BMI, potentially suggesting brain-based metabolic susceptibility for a higher BMI, therefore for higher weight. Conversely, a negative BMIgap indicates that an individual's brain resembles that of someone with lower BMI than their actual BMI, potentially suggesting brain-based atypical metabolic processes for a lower BMI, therefore for lower weight. Thus, this metric provides a personalized, brain-derived measure of BMI deviation and, in consequence, reflects brain-based vulnerability toward BMI-related metabolic pathophysiology.

The model was first validated in two independent healthy control samples, $HC_{validation}$ and $HC_{Cam\text{-}CAN}$, to assess its generalizability. We then applied the BMI predictor to clinical populations including individuals with schizophrenia, ROD and CHR. By comparing their BMIgap scores to the reference ($HC_{discovery}$), we assessed whether these groups exhibit systematic deviations of brain-based BMI patterns.

Machine learning analysis

To prevent information leakage between train and test data and enhance model generalizability, we implemented a repeated nested cross-validation (CV) cycle with 5 folds at inner (CV1) and outer CV cycles (CV2) each, and 5 permutations of both cycles (which produced 625 CV1 training and test partitions in total). The hyperparameter optimization was confined to the inner folds, effectively preventing overfitting while optimizing the model.

Data pre-processing involved the following pre-processing steps: (i) Gaussian smoothing with 0, 3, 6 and 9 mm full-width-at-half maximum (FWHM) kernel widths, (ii) regressing out age effects using partial correlation analysis, (iii) mean offset correction to remove site effects, (iv) principal component analysis (PCA) with different PCA energy levels (0.25, 0.50, 0.75) to reduce the dimensionality of the image space, and (v) voxel-wise scaling from 0 to 1. All the pre-processing steps were fully wrapped within each training cycle. Further, the model used a linear kernel type nu-support vector machine regression algorithm with a regularization parameter of 1. We measured the precision of the model's BMI estimates in the CV1 test and CV2 validation data, using the mean absolute error (MAE), Pearson's correlation coefficient (r) and the coefficient of determination (R^2) between individual observed BMI and predicted BMI. The MAE was selected as the optimization criterion. Optimization consisted of finding the hyperparameter combination amongst 4 (smoothing) × 3 (PCA) × 1 (nu) = 12 hyperparameter combinations that maximized the average MAE computed across the CV1 test data partition (detailed in Figure S2 and Supplementary results). Models were retrained using the CV1 training and test data at the optimal hyperparameter combination before being applied to the CV2 validation data. The final BMI prediction of a CV2 individual was computed by averaging the predictions of those models, which did not use the given person for training of hyperparameter optimization. The BMI prediction model's statistical significance was evaluated using 1000 random BMI label permutations and determined at $\alpha=0.05$.

Predictive brain patterns were visualized using the grand-mean of cross-validation ratio (CVR) as a measure of feature stability (3) and a sign-based consistency metric assessing feature importance (12). To retain only significant voxels, we thresholded the CVR map based on the false-discovery rate (FDR)-corrected sign-based consistency map (α=0.05). The open-source 3-dimensional rendering software MRIcroGL (McCausland Center for Brain Imaging, University of South Carolina; https://www.nitrc.org /projects/mricrogl/) was used to overlay the thresholded map on the Montreal Neurological Institute (MNI) template to produce 3-dimensional renderings and axial mosaic slices. Additionally, the Automated Anatomical Labelling (AAL3) atlas was overlaid to visualize predictive regions-of-interest (ROIs) (13) in a spider-plot. Then, we applied the discovery model to the independent validation sample to assess the model's generalizability. Further, the discovery model was applied to the SCZ, ROD and CHR patients to obtain brain-based BMI predictions. The HC individuals' and patients' BMIgap was calculated by subtracting the original from the predicted BMI scores.

Site-correction

Previous studies have shown that structural magnetic resonance imaging (sMRI) data is susceptible to site or scanner effects (14,15). We used the data from four cohorts and two of which are multi-site studies. Considering this, we applied a global mean correction procedure within the nested cross-validation settings to remove the site effects. This involves subtracting the mean value of each site from the overall mean and then subtracting this mean difference from each feature of the other sites.

Leave-site-out cross-validation

To test the geographic generalizability a nested leave-one-site-out cross-validation (LOSOCV) was implemented. The discovery model, using the same preprocessing steps, was trained within a LOSOCV framework, where each study site was iteratively held back as validation fold in

the outer loop (16). A 5-fold pooled cross-validation procedure was conducted in the inner CV cycle to optimize model hyperparameters, ensuring that parameter tuning was strictly separated from model validation, thus preventing any site-related information leakage.

Post-hoc BMIgap correction for true BMI

To remove the effects of BMI from BMIgap for discovery, validation, and clinical groups we followed the stepwise calibration strategy: (i) We implemented the conceptual idea of k-fold cross validation. We split the discovery sample into five smaller groups. In each iteration, one-fold was a hold-out set (equivalent to the test-set) and the remaining samples (equivalent to the training group) were considered for calculating the beta values using partial correlation. (ii) the calculated beta coefficient was applied to the hold-out fold of the discovery sample, the whole validation sample, and the clinical groups to obtain the corrected BMIgap values. This was repeated until all folds have been used as the hold-out test-set once. Finally, we calculated the mean BMIgap across all folds to find a corrected BMIgap score for each individual. Comparision of corrected and uncorrected BMIgap is depicted in Figure S3-S4.

Classification model for HC/schizophrenia

We created an sMRI-based classification model within NeuroMiner that distinguished individuals with schizophrenia from HC to extract schizophrenia-specific brain patterns. We used the following pre-processing steps: (i) Gaussian smoothing with 0, 3, 6 and 9 mm full-width-at-half maximum (FWHM) kernel widths, (ii) regressing out age effects using partial correlation analysis, (iii) principal component analysis (PCA) with different PCA energy levels (0.25, 0.50, 0.75) to reduce the dimensionality of the image space, and (iv) voxel-wise scaling from 0 to 1 similar to the model parameters used in the BMI prediction model within a repeated nested CV cycle with 5 folds at inner and outer cycles each, and 5 permutations for both cycles, like for the BMI regression model. Additionally, we used a GMV specific brain mask to retain

only voxels specific to the GMV regions. Further, the model used a linear class-weighted support vector machine (SVM) as classification algorithm with optimization performed for the regularization parameter C_{SVM} over the range of $2^{[-4 \rightarrow +4]}$ (11 parameters). We used sensitivity, specificity, accuracy, and balanced accuracy (BAC) as metrics to evaluate the performance of this model at the CV1 test and CV2 validation datasets. BAC was selected as optimization criterion.

$$BAC=(Sensitivity+Specificity)/2$$

Optimization consisted of finding the hyperparameter combination amongst 4 (smoothing) × 3 (PCA) × 11 (C_{SVM}) = 132 hyperparameter combinations maximizing the BAC across CV1 test data partition. We visualized the predictive voxels using the sign-based consistency metric as described earlier.

Model Visualization

We evaluated the statistical significance of classification/regression model performances by comparing the observed respective optimization criterion BAC/*r* with an empirical null-distribution of the respective out-of-training BAC/*r* obtained by permuting the group labels 1000 times and retraining the models within the cross-validation scheme. Afterwards, we computed the probability of the observed BAC/*r* as the number of cases in which the permuted BAC/*r* was equal or higher than the observed BAC/*r* divided by 1000 and evaluated statistical significance at α=0.05, using FDR correction to control for multiple comparisons. For the visualization of the predictive features, we primarily employed a measure of feature stability termed grand mean CVR (described in detail in Koutsouleris et al., 2021 (17) and adapted from Krishnan et al., 2011 (18)). This is calculated as the sum of the selected CV1 median weights across all the CV2 folds divided by the standard error of the selected CV1 weights for each feature, similar to the bootstrap ratio (BSR) approach in partial least squares (PLS) studies

(18,19). The BSRs show how reliably each source is contributing to the observed pattern. A BSR thresholded at ±2 is considered stable. Additionally, we computed a metric of feature importance called sign-based consistency, which assigns feature relevance based on the times that a specific feature has the same sign (positive/negative) across the ensemble, adapted from the method of Gómez-Verdejo et al., 2019 (12) and detailed in Koutsouleris et al., 2021 (17). For visualizing the predictive brain voxels in the BMI-predicting model for the discovery sample: (i) we binarized the sign-based consistency maps to retain the significant voxels thresolded at α=0.05, and (ii) we multiplied the CVR with the binarized sign-based consistency maps. The resulting map showed the significant stable CVR with the warm and cool colored regions differentiating regions with positive and negative correlation of GMV and estimated BMI, respectively.

Overlapping brain regions

To identify shared brain regions predictive of both BMI and schizophrenia, we followed the following steps: (i) we binarized the FDR-corrected (α=0.05) sign-based consistency maps which were generated during the model visualization step for the regression and classification models and retained only the significant voxels ($P>0.001$), and (ii) created the overlap between the two binarized maps, resulting in a brain-mask that specifically represents the overlapping regions predictive of BMI and schizophrenia (Figure S7).

Investigation of phenotypic BMIgap associations

To investigate the phenotypic associations between obesity, schizophrenia and clinically relevant variables we employed multivariate Sparse Partial Least Squares Analysis (SPLS) as implemented in the SPLS Toolbox by Popovic et al. 2020 (20) in which this algorithm is embedded in a nested CV framework with five folds each on the outer CV2 and the inner CV1 cycle. The schizophrenia subjects who had less than 30% missing values in the clinical

variables (N=139) were included in the SPLS analysis to detect the associations between two data domains: (i) the brain data matrix consisting of the overlapping GMV regions between the significant voxels produced from the BMI-prediction model and the classification model, and (ii) five feature matrix including clinical dimensions such as BMIgap values, schizophrenia expression-score and clinical items such as PANSS total score, age of disease onset, illness duration and the number of hospitalizations. The SPLS algorithm uses singular value decomposition to generate latent variables (LV), which represent distinct, multivariate associative effects between the two data matrices. A LV consists of a weight vector pair (*u, v)*, which places weights on individual features in the respective brain and clinical data matrix. Here, *u* represents the brain pattern and *v* the clinical-dimension pattern of the LV with the feature weights varying between -1 to 1, indicating the direction and strength of covariance between the respective features. Furthermore, two feature weights (*u, v*) having the same signum (i.e., both positive or both negative) for the respective features covary positively with each other, two feature weights with opposite signum for their feature weights have a negative covariation with each other and, finally zero weighting is indicative of non-significant contribution of the feature to the respective covariance signature. Additionally, the two weight vectors form a new latent space, in which individuals are represented by their respective latent scores as calculated by multiplying individualized clinical and brain data with the respective clinical and brain vectors of the LV, thereby obtaining two numerical values, representing participant's individual loadings onto these weight vectors. The correlation between the latent scores for all individuals indicates the effectiveness of the weight vector pair of the LV to maximize the covariance. The significance of the associative effects from the SPLS analysis were determined using 5000 random permutations of the brain-clinical design matrix, followed by bootstrap resampling to identify stable brain-clinical features in the significant LVs. Finally, significant patterns were mapped to the 17-network parcellation solution of Yeo-Buckner atlas

for visualization (21). The network names and the cerebral cortical regions that compose the 17 networks are from the supplementary video in Baker et al. (2014) (22). The BSRs are plotted separately for clinical and brain-based findings. The significance of the associative effects was determined using 5000 random permutations of the brain-behavioral design matrix, followed by bootstrap resampling (N=500) to identify the most stable features within the respective LVs. Specifically, we used BSR, calculated as the ratio between each variable's bootstrap-estimated mean weight to its standard error, to quantify the reliability of each variable within the overall brain-behavioral pattern of the LV (23). Only feature weights with a BSR>|2| were considered as reliably contributing to the respective LV.

Machine learning analysis to predict future weight-change

We conducted a multivariate classification to categorize subjects with 3%, 5%, and 7% weight-changes, using BMIgap and selected clinical variables. The ROD and CHR subjects who had less than 30% missing values as well as the clinical items which had less than 30% of missing values were included in this analysis. The final clinical variables were BMIgap, age, sex, study group (ROD, CHR), exercise (strenuous exercise or mindfulness activities such as yoga and meditation), and history of somatic comorbidities (i.e. whether the individual suffered from somatic illness) as features. These items were used as features to classify whether subjects underwent future weight gain. The pre-processing steps included imputing missing values, regressing out study group information, and standardizing the data. Further, the model used a linear class-weighted SVM as classification algorithm with optimization performed for the regularization parameter C_{SVM} over the range of $2^{[-4 \rightarrow +4]}$ (11 parameters). We used sensitivity, specificity, accuracy, and BAC as metrics to evaluate the performance of this model at the CV1 test and CV2 validation datasets. The analysis was performed within a nested CV framework with 5 folds for CV1, 5 folds for CV2 with 5 permutations for each cycle.

Weight change and clinical outcomes

We examined the association between weight gain (ΔW1 and ΔW2) and clinical measures, including global functioning (GAF; symptom and disability subscales), as well as symptom severity (PANSS; positive, negative, and general subscales), and depressive symptoms (BDI) at T1 and T2. Additionally, we examined the relationship between weight gain and total medication dosage. Substances were further categorized into weight-gain-associated and weight-neutral classes based on established literature, and correlations between dosage and weight gain were analyzed separately for each category.

Supplementary Results

Hyperparameter optimization

The BMI-predictor opted for a 3 mm FWHM Gaussian smoothing kernel with the highest selection frequency across the folds in the support vector subspace. Moreover, the discovery model optimized for 0.75 PCA energy level.

The classification model also opted for the 3 mm FWHM Gaussian smoothing kernel with the highest selection frequency across the folds in the support vector subspace and optimized for 0.75 PCA energy level.

Clinical associations in brain regions predictive of BMI and schizophrenia

The SPLS analysis yielded five significant LVs, representing distinct levels of association between the neuroanatomic overlap regions of the BMI and schizophrenia models and the clinical disease features (Figure 2, Figure S8). LV1 and LV4 extracted covariate patterns of age and sex (Supplement Results).

In LV1 (r=0.87, P<0.001), lower BMIgap scores and higher schizophrenia expression-scores were related to decreased GMV in the ventral attention network and increased GMV in the default mode network (DMN) (auditory), somatomotor-B, control-B/C, and central visual networks (Figure S8a).

In LV4 (r=0.44, P=0.025), higher schizophrenia expression-scores, illness duration, age of onset, and number of hospitalizations and lower BMIgap and PANSS total scores were related to decreased GMV in the limbic as well as Control-A/B , DMN-A/B, somatomotor-A/B, salience, dorsal and ventral attention, and peripheral visual networks and increased GMV in non-overlapping subcomponents DMN-C and control-C networks (Figure S8b).

Association between weight change and clinical outcomes

Associations between weight gain and clinical outcomes revealed that greater weight gain between T0 and T1 (ΔW1) was significantly associated with higher PANSS-N (r = 0.186, P = 0.001, N = 289) and PANSS-G (r = 0.203, P = 0.001, N = 289) at T1. Additionally, weight gain between T0 and T2 (ΔW2) correlated negatively with GAF:S (r = -0.229, P = 0.002, N = 174) and GAF:D/I (r = -0.144 to -0.152, $P < 0.05$). No significant associations were found between weight gain and PANSS-P or BDI-II scores at any timepoint. Medication dosage analysis showed no significant association between overall medication dosage and weight gain (T1: r = 0.065, P = 0.376, N = 190; T2: r = 0.061, P = 0.526, N = 110). However, when stratified by medication type, individuals receiving weight-gain-associated medication showed a significant correlation with weight gain at T1 (r = 0.217, P = 0.023, N = 110), but not at T2 (r = 0.181, P = 0.159, N = 62). Weight-neutral medication did not show significant associations with weight gain at either timepoint (T1: r = -0.126, P = 0.402, N = 46; T2: r = -0.269, P = 0.143, N = 31) (Table S5-S6).

Supplementary Discussion

Our findings indicate that both psychotic illness severity and functioning (PANSS and GAF) as well as exposure to weight-gain-associated medication contributes to weight trajectories in early-stage psychiatric populations. Higher ΔW1 was significantly associated with higher PANSS negative and general symptoms and lower GAF scores, highlighting the role of

functional and symptomatic burden in metabolic vulnerability. These associations were still evident, though attenuated, at two-year follow-up (ΔW2). In contrast, depressive symptom severity (BDI) showed no significant association with weight gain, suggesting that core depressive symptoms may not be the primary drivers of early metabolic changes. Additionally, higher dosages of weight gain-associated medication were associated with increased early weight gain, likely reflecting short-term pharmaco-metabolic effects during acute treatment. This association did not persist at T2, potentially due to smaller sample sizes or physiological adaptation over time. Weight-neutral medication was not associated with increased weight gain, and trends suggested a possible stabilizing role. Together, these findings highlight the interplay between clinical severity and pharmacological treatment in shaping early weight trajectories and reinforce the need for early, possibly neurobiologically guided, metabolic monitoring—especially during the first year of treatment—in vulnerable psychiatric populations.

Supplementary Figures

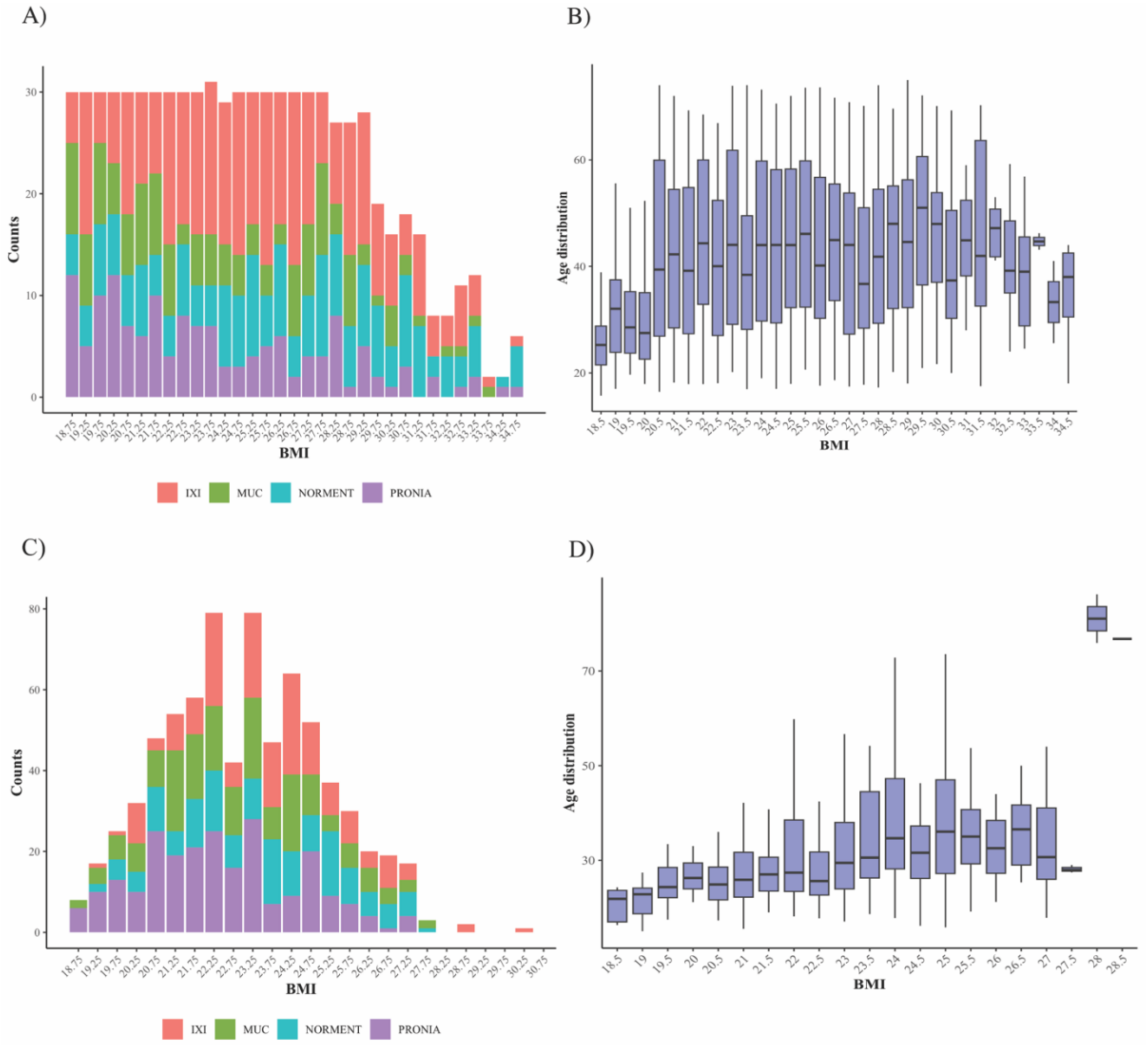

Figure S1. BMI and age distributions for discovery (N=770) and validation (N=734) sample. Histogram for the BMI distribution for A) discovery showing the uniform-like distribution, and C) validation sample with the different colors representing the four cohorts. Age distribution per BMI bin of 0.5 BMI units for B) discovery and D) validation sample.

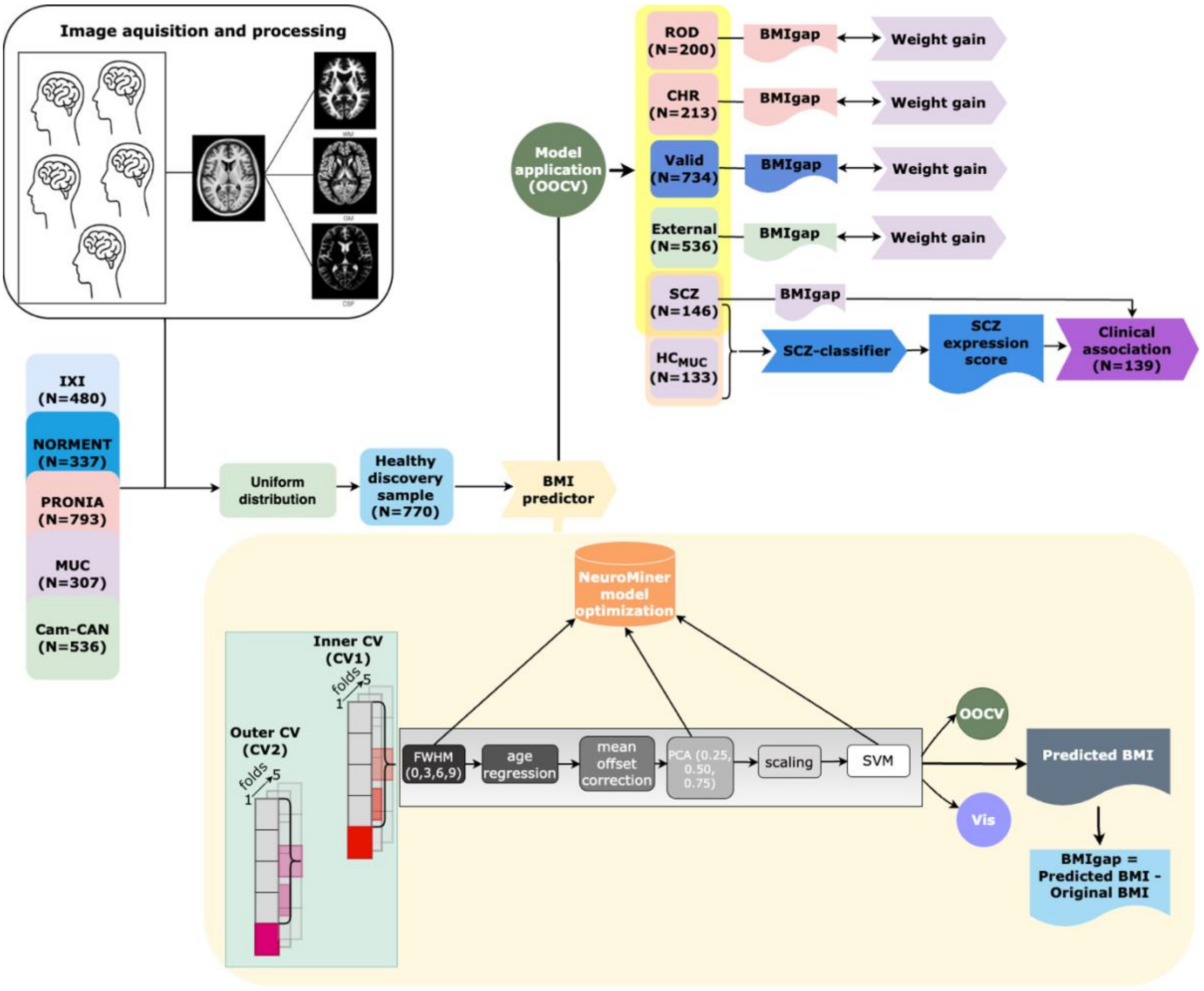

Figure S2. Schematic representation of analysis flow conducted in the study. Abbreviations: HC= healthy control individuals, MUC= Munich database, BMI= Body mass index, ROD= Recent onset depression, CHR= Clinical high risk, SCZ= Schizophrenia, SPLS= Sparse partial least squares, CV= Cross validation, PCA= Principal component analysis, SVM= Support vector machine, Vis= Model visualization, OOCV= Out-of-sample cross-validation

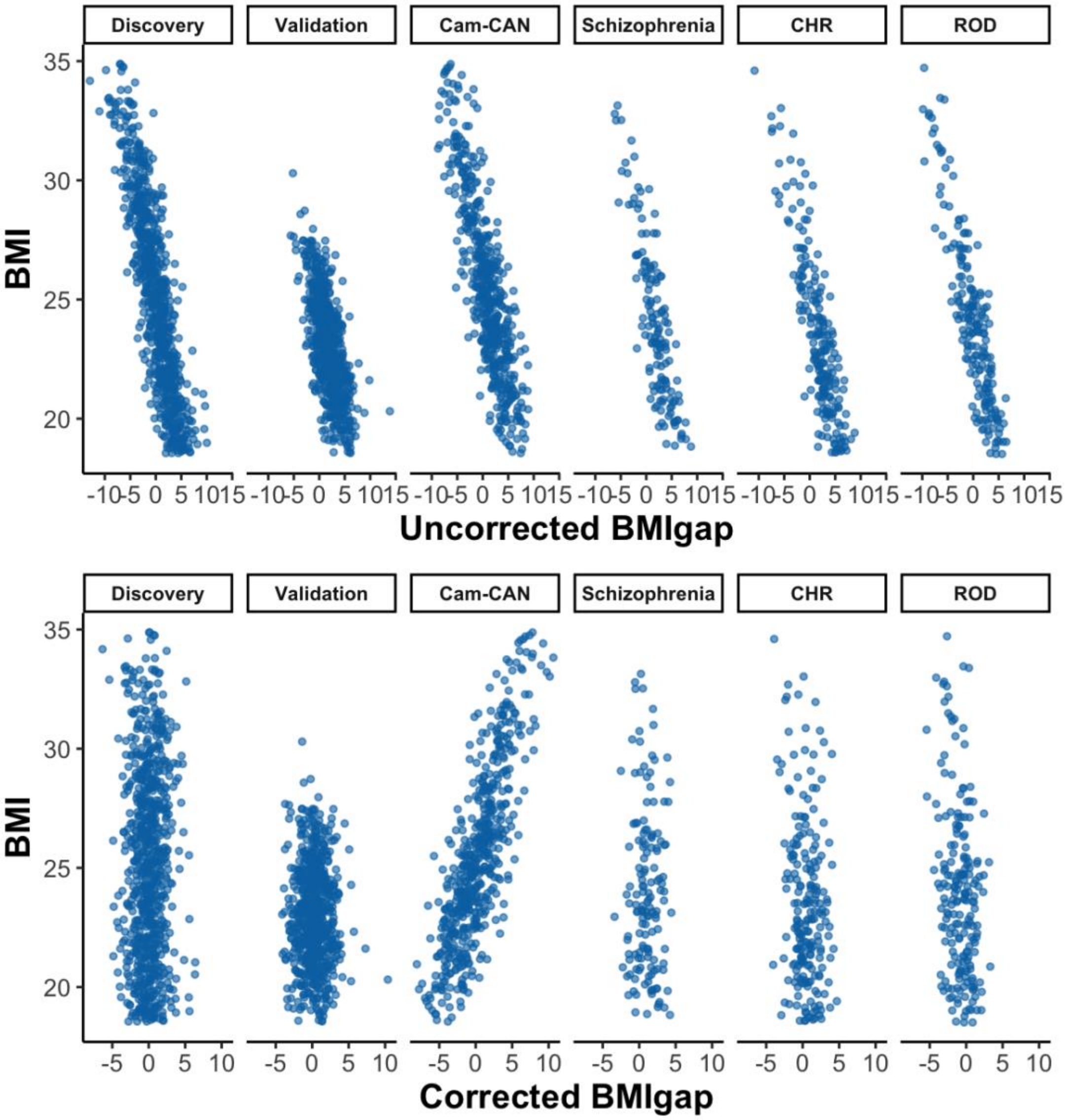

Figure S3: Scatter plots for BMIgap before and after the BMIgap correction for BMI for the discovery, validation, and clinical groups. Abbreviations: BMI= Body mass index, ROD= Recent onset depression, CHR= Clinical high risk

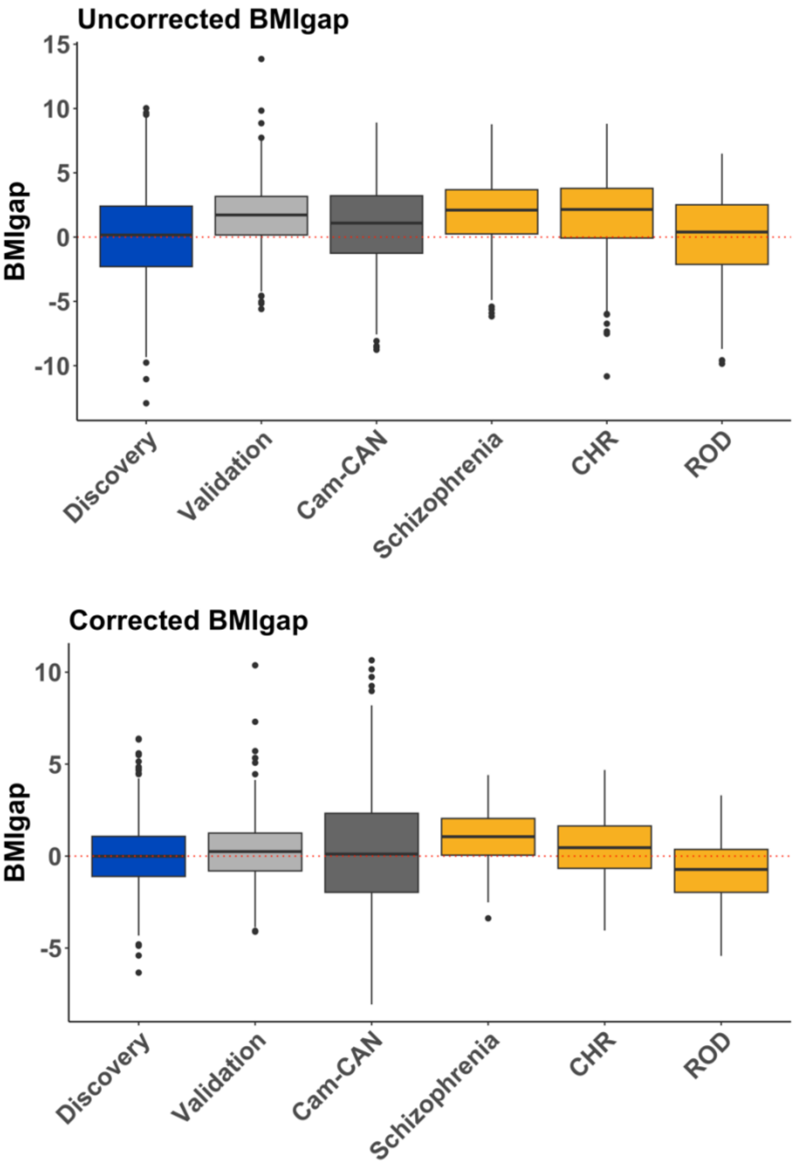

Figure S4: Group-level BMIgap analysis. BMIgap distributions across clinical groups are visualized as box plots. BMIgap are visualized before and after the correction of BMIgap for BMI. Abbreviations: BMI= Body mass index, ROD= Recent onset depression, CHR= Clinical high risk

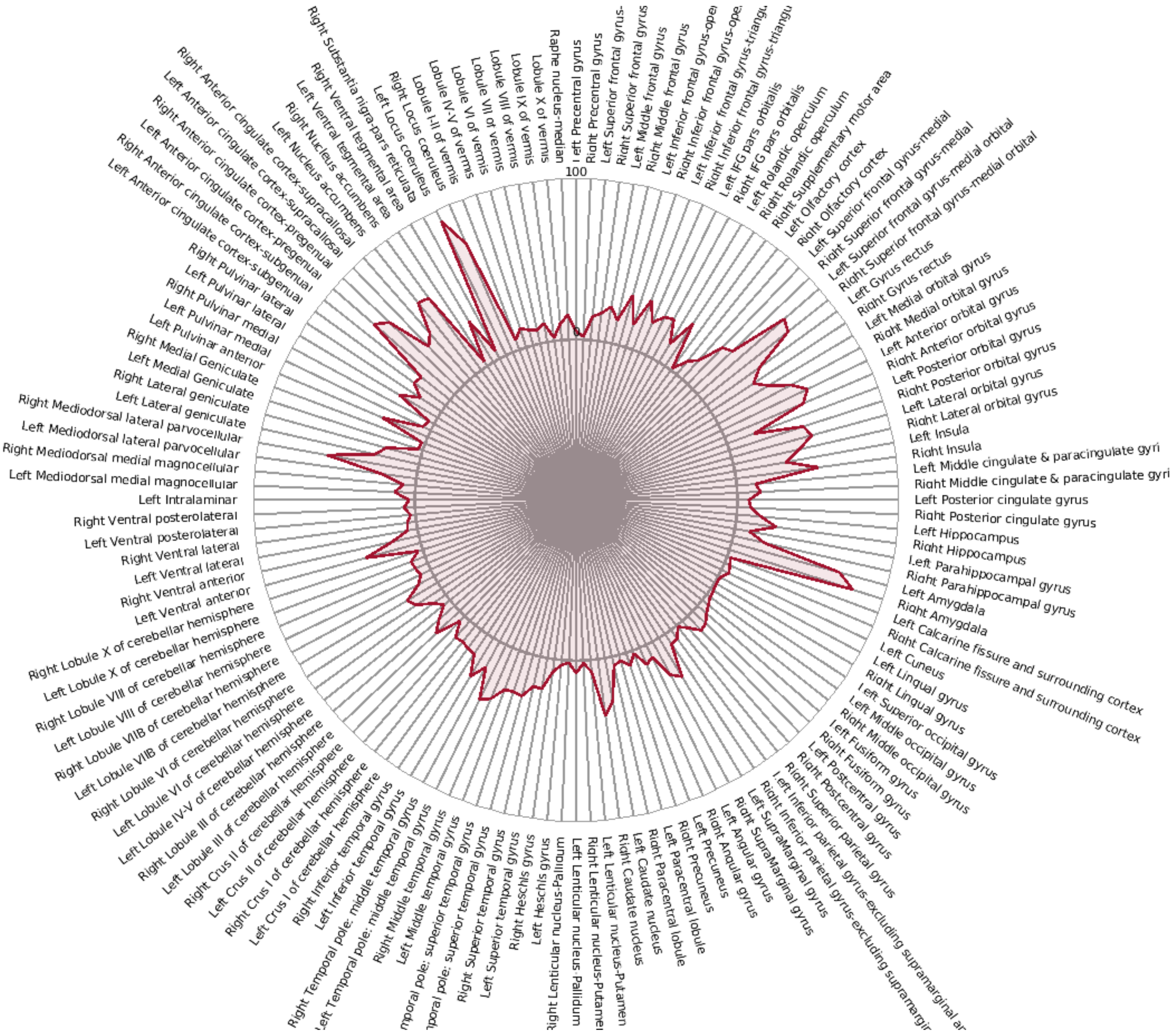

Figure S5: Regional distribution of predictive brain-based BMI patterns of the discovery model. The percentage of voxels occupied by the predictive patterns was quantified based on the AAL3 atlas (13).

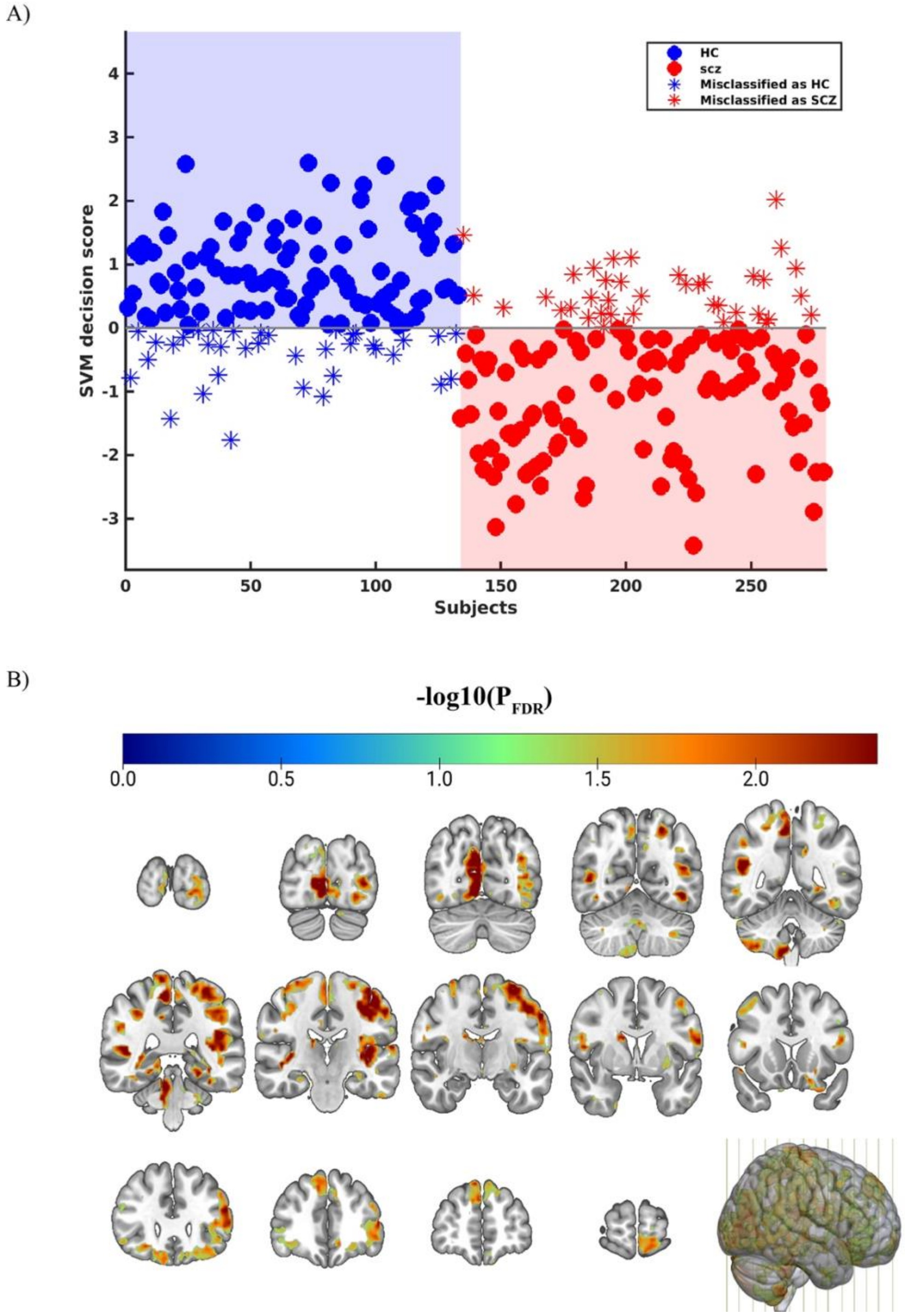

Figure S6: Classification model for classifying patients with schizophrenia (N=146) from HC individuals (N=133). A) Classification performance. B) The reliability of the predictive voxels visualized using FDR-corrected sign-based consistency map thresholded at α=0.05. Abbreviations: FDR= False discovery rate

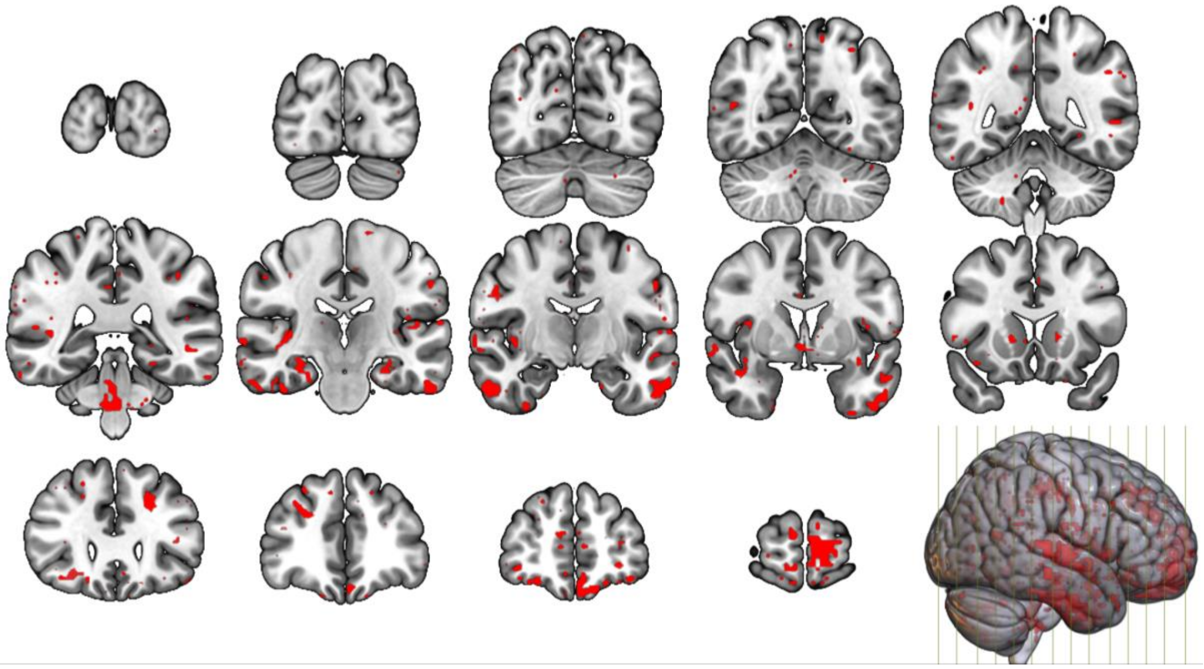

Figure S7: Overlapping brain regions. Overlapping regions across schizophrenia and obesity obtained by binarizing and multiplying the sign-based consistency maps from the regression and classification models (α=0.05).

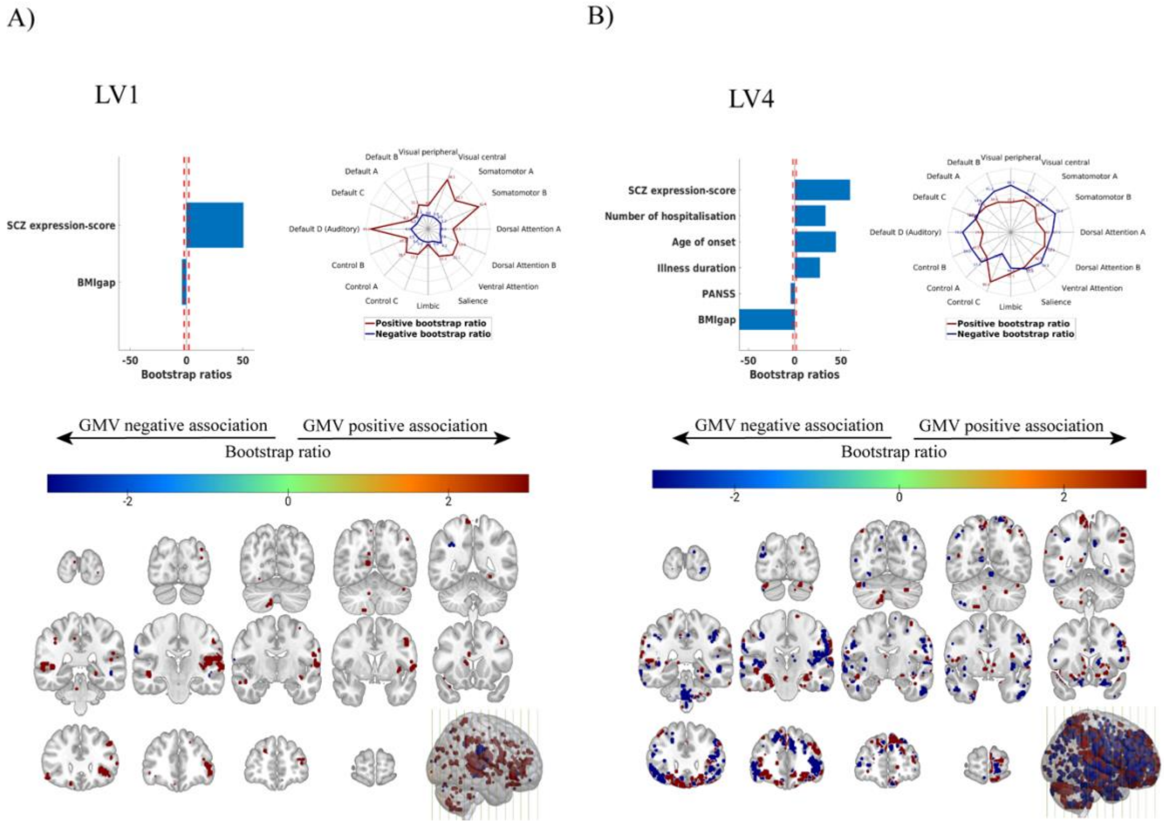

Figure S8: Signature for schizophrenia and BMIgap effects. SPLS analysis results for A) LV1 and B) LV4. Bar plots visualize the correlation of each variable with the LV, blue identifies variables significantly contributing to the LV. The x-axis denotes bootstrap ratios (BSR) (interpretable as z-scores) and the y-axis denotes BMIgap, schizophrenia expression-score and other clinical items. The red dotted line in the graph represents a BSR of 1.96 (equivalent to a 95% confidence interval). The contribution of individual voxels is shown using BSR in MNI space. Cool colors indicate voxels with a negative correlation of GMV and clinical items, whereas warm colors represent a positive correlation. The spider-plot represents the voxel contribution within the 17-network parcellation solution of the Yeo-Buckner atlas (21). The network names and the cerebral cortical regions that compose the 17 networks are from the supplementary video in Baker et al. (2014) (22). Abbreviations: LV= Latent variable, SCZ= Schizophrenia, BMIgap= body mass index gap score, PANSS= Positive and Negative Symptom Scale total score

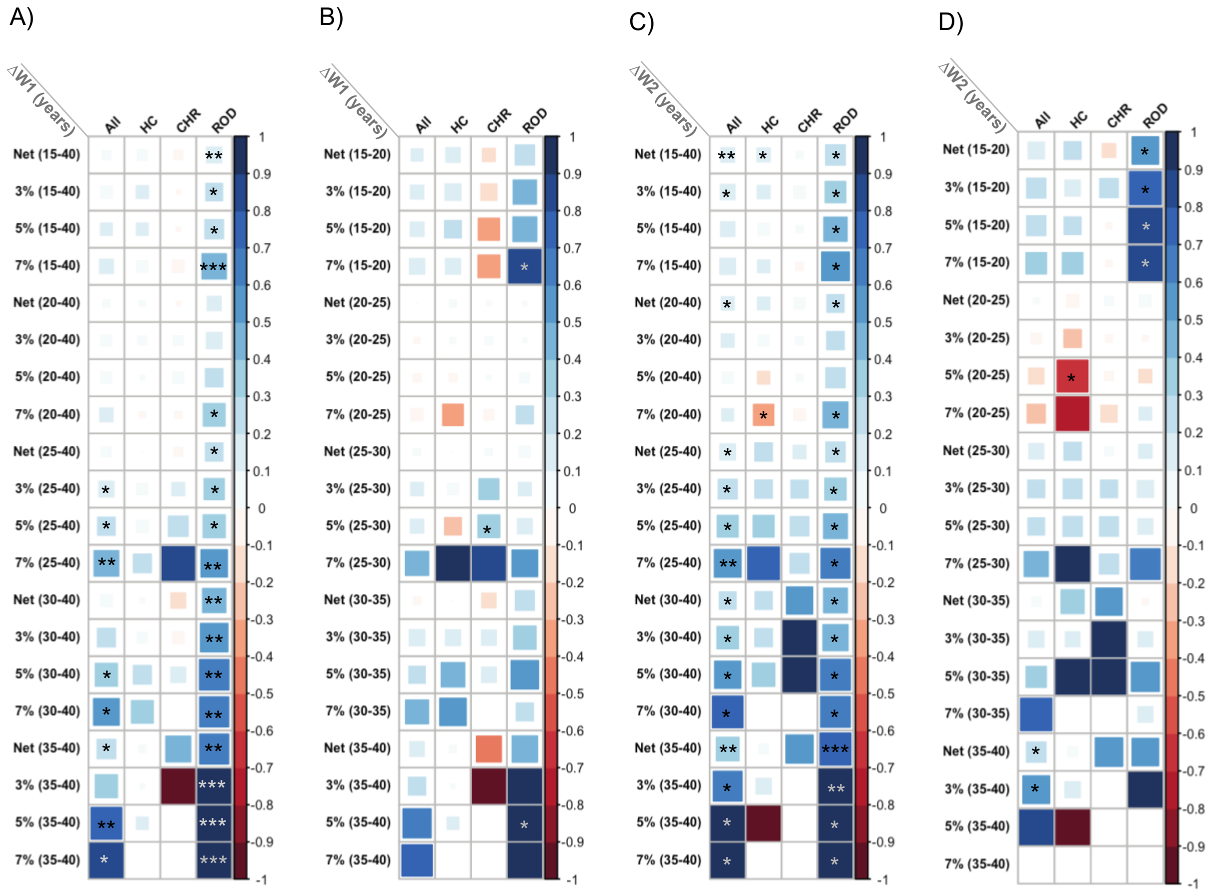

Figure S9: Correlation between BMIgap and weight change at 1- and 2-year follow-up. Heatmap showing correlations between BMIgap and weight change across different age ranges and clinical groups (HC, CHR, ROD). Correlation coefficients were calculated using two-sided Pearson correlations. Significance was assessed with FDR correction for multiple comparisons; significant values are denoted as *P ≤ 0.05, **P ≤ 0.01, **P ≤ 0.001, with exact (FDR-adjusted) P values reported in Tables S7–S10. The plot uses blue squares to represent positive correlations and red squares for negative correlations. The size of each square corresponds to the magnitude of the correlation value, with larger squares indicating stronger correlations. The correlations across different age windows including A), C) 15-40, 20-40, 25-40, 30-40 years and as well as within 5-year bins B), D) 15–20, 20–25, 25–30, 30–35, and 35–40 years. Abbreviations: ΔW_1= weight at T1 – Weight at T0, ΔW_2= Weight at T2 – Weight at T0, BMIgap= body mass index gap score, HC=Healthy controls, CHR= Clinical high-risk, ROD= Recent-onset depression, Net= any weight change without defining a threshold.

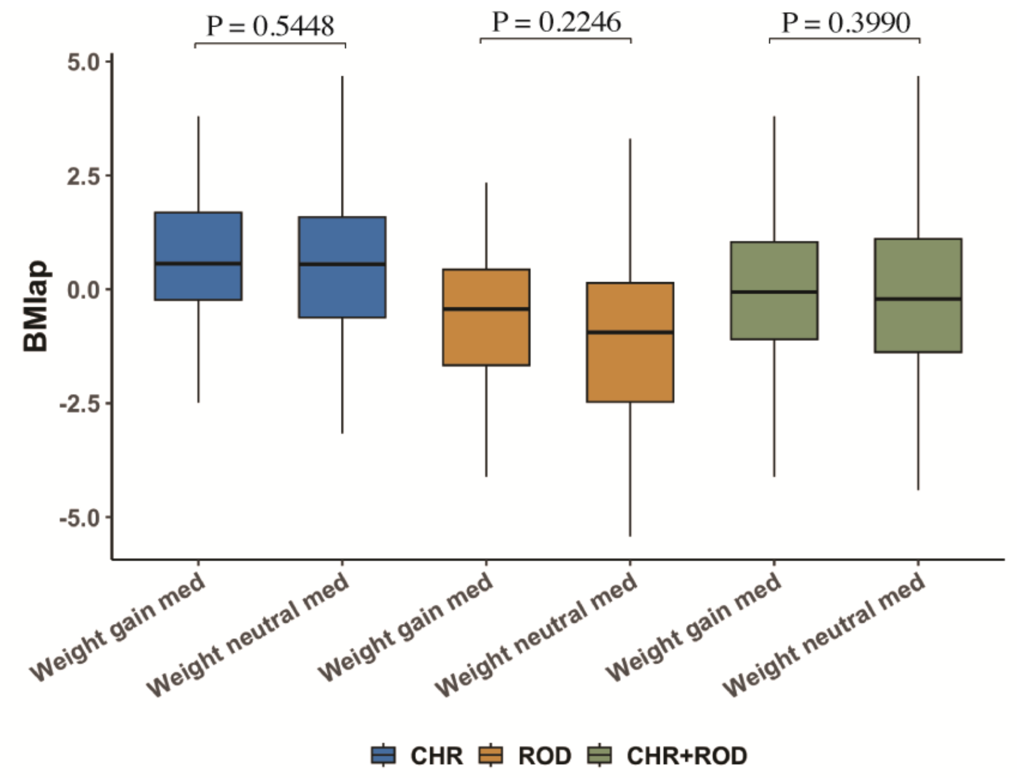

Figure S10: BMIgap by medication-associated weight gain across clinical groups. Boxplots show BMIgap values stratified by clinical group (CHR, ROD) and medication category: weight-gain–associated medication (CHR: N=81; ROD: N=92; CHR+ROD: N=173) versus weight-neutral/no medication (CHR: N=28; ROD: N=20; CHR+ROD: N=48). Comparisons were performed using two-sided paired t-tests: CHR, t=0.6068, df=107, P=0.5448; ROD, t=1.2187, df=110, P=0.2246; combined CHR+ROD, t=0.8445, df=219, P=0.3990.Abbreviations: BMIgap= body mass index gap score, CHR= Clinical high-risk, ROD= Recent-onset depression, P=P value.

Supplementary Tables

Table S1: MRI scanner systems and structural MRI sequence parameters used in the respective cohorts.

Abbreviations: TR = repetition time, TE = echo time, FOV = field of view.

Site	Model	Field Strength	Coil Channels	Flip Angle	TR [ms]	TE [ms]	Voxel Size [mm]	FOV	Slice Number
IXI database									
Hammersmith Hospital	Philips Intera	3.0T	-	8	9.6	4.6	-	208 x 208	-
Institute of Psychiatry	Philips Gyroscan Intera	1.5T	-	-	-	-	-	-	-
Guy's Hospital	General Electric	1.5T	-	8	9.8	4.6	-	208 x 208	-
NORMENT									
Oslo	SIEMENS Magnetom	3T	32	7	2730	3.93	1.33 × 0.94 × 1.0	240 x 240	160
Munich database									
LMU Hospital	SIEMENS Magnetom	1.5T	8	12	11.6	4.9	0.45 x 0.45 x 1.5	230 x 230	126
PRONIA									
Munich	Philips Ingenia	3T	32	8	9.5	5.5	0.97 x 0.97 x 1.0	250 x 250	190
Milan Niguarda	Philips Achieva Intera	1.5T	8	12	Shortest (8.1)	Shortest (3.7)	0.93 x 0.93 x 1.0	240 x 240	170
Basel	SIEMENS Verio	3T	12	8	2000	3.4	1.0 x 1.0 x 1.0	256 x 256	176
Cologne	Philips Achieva	3T	8	8	9.5	5.5	0.97 x 0.97 x 1.0	250 x 250	190
Birmingham	Philips Achieva	3T	32	8	8.4	3.8	1.0 x 1.0 x 1.0	288 x 288	175
Turku	Philips Ingenuity	3T	32	7	8.1	3.7	1.0 x 1.0 x 1.0	256 x 256	176
Udine	Philips Achieva	3T	8	12	Shortest (8.1)	Shortest (3.7)	0.93 x 0.93 x 1.0	240 x 240	170
Muenster	Siemens Magnetom PRISMA-FIT	3T	20	8	2130	2,28	1x1x1	256	192
Duesseldorf	Siemens Prisma	3T	32	8	2000	3.37	1.0x1.0x1.0	256x256	176
Bari	Philips Ingenia	3T	32	8	8.1	3.7	1.0 x 1.0 x 1.0	256 x 256	180
Cam-CAN									
Cambridge	Siemens TIM Trio	3T	-	9	2250	2.99	1.0 x 1.0 x 1.0	256 x 240 x 192	-

Table S2: Inclusion and exclusion criteria for the PRONIA HC. Adapted from Koutsouleris et al. 2018 (3)

Group	Inclusion Criteria	Exclusion Criteria
Healthy Controls		1. Any current or past DSM-IV axis disorder<br>2. A positive familial history (1st degree relatives) of affective or non-affective psychoses or major affective disorders;<br>3. Intake of psychotropic medication or drugs more than 5 times/year or in the month before study inclusion.
General	1. Age between 15 and 40 years<br>2. Sufficient language abilities for participation<br>3. Ability to provide consent / assent	1. IQ below 70<br>2. Hearing is not sufficient for neuro-cognitive testing<br>3. Current or past head trauma with loss of consciousness (> 5 min)<br>4. Current or past known neurological disorder of the brain<br>5. Current or past known somatic disorder potentially affecting the structure or functioning of the brain<br>6. Current or past alcohol dependence<br>7. Current poly-substance dependence or within the past six months<br>8. Any contra-indication for MRI

Table S3: BMI-predictive voxels. The voxels occupied by the BMI-predictive patterns were quantified based on the AAL3 atlas (13).

Volume (mm^3)	Peak Intensity value	Peak (x, y, z)	Peak Structure	Structure (according to the AAL3 atlas)
10007	4.8	0.2×-36.3×-36.4		-(87) Cerebelum_8_L(7) Cerebelum_9_L(2) Cerebelum_10_L(2) Cerebelum_10_R(1) Vermis_10 (1)
6087	4.2	33.4×-3.1×35.9		-(97) Precentral_R(2) Putamen_R(1)
322	4.1	14.9×-48.1×24.1	Precuneus_R	-(51) Precuneus_R(36) Cingulum_Post_R(13)
7016	3.7	-24.1×-11.9×35.9		-(91) Thalamus_L(5) Insula_L(2) Rolandic_Oper_L(1)
441	3.7	42.2×-23.7×30.0		-(95) Rolandic_Oper_R(5)
360	3.7	39.3×-39.2×21.2		-(97) Temporal_Mid_R(3)
937	3.6	-33.0×-29.6×0.5		-(79) Thalamus_L(13) Hippocampus_L(8)
244	3.6	-11.6×-48.1×21.2	Cingulum_Post_L	Precuneus_L(53) Cingulum_Post_L(26) -(21)
630	3.5	14.9×-9.0×-5.4		-(77) Thalamus_R(19) Pallidum_R(4)
117	3.5	-47.7×-29.6×-17.9	Temporal_Inf_L	Temporal_Inf_L(47) Temporal_Mid_L(37) -(15)
779	3.4	26.7×-26.7×-0.2		-(94) Thalamus_R(5) Pallidum_R(1)
62	3.3	48.1×-0.1×18.2	Rolandic_Oper_R	Rolandic_Oper_R(59) -(41)
1312	3.3	14.9×-59.9×-39.3	Cerebelum_8_R	-(52) Cerebelum_8_R(40) Vermis_9(5) Cerebelum_9_R(3)
115	3.3	-41.8×-45.1×15.3		-(99) Temporal_Mid_L(1)
138	3.3	29.7×-93.1×-6.1	Occipital_Inf_R	Occipital_Inf_R(100)
140	3.2	-38.9×-23.7×-9.1		-(87) Hippocampus_L(13)
33	2.9	42.2×-18.6×-12.0		-(67) Hippocampus_R(33)
89	2.8	-8.7×-3.1×59.5	Supp_Motor_Area_L	Supp_Motor_Area_L(60) -(38) Frontal_Sup_L(2)
57	2.7	0.2×-36.3×15.3		-(99) Cingulum_Post_R(1)
80	2.6	-9.4×-9.0×27.1		-(100)
45	2.5	-3.5×-41.4×14.5		-(67) Cingulum_Post_L(31) Precuneus_L(2)
49	2.4	-38.9×-32.6×-36.4	Cerebelum_6_L	Cerebelum_Crus1_L(36) -(35) Cerebelum_7b_L(23) Cerebelum_6_L(7)
38	-2.3	-18.2×-14.9×-32.7		-(82) ParaHippocampal_L(18)
115	-2.4	6.1×-90.1×-32.7	Cerebelum_Crus2_R	Cerebelum_Crus2_R(76) -(24)
44	-2.4	-11.6×-26.7×0.5	Thalamus_L	Thalamus_L(95) -(5)
108	-2.4	-66.2×-11.9×14.5	Postcentral_L	-(72) Postcentral_L(21) Rolandic_Oper_L(3) Temporal_Sup_L(3) Heschl_L(1)
72	-2.4	57.0×-51.0×-20.9	Temporal_Inf_R	Temporal_Inf_R(100)

68	-2.5	45.2×-56.9×56.6	Parietal_Inf_R	Parietal_Inf_R(82) Angular_R(18)
35	-2.5	35.6×-45.1×-51.1	Cerebelum_8_R	Cerebelum_8_R(100)
183	-2.5	17.9×-51.0×-54.1	Cerebelum_8_R	Cerebelum_8_R(78) Cerebelum_9_R(22)
257	-2.5	17.9×-65.8×66.1	Parietal_Sup_R	Parietal_Sup_R(100)
44	-2.5	-27.1×-68.7×-17.9	Cerebelum_6_L	Cerebelum_6_L(100)
51	-2.6	9.0×-24.5×47.7	Cingulum_Mid_R	Cingulum_Mid_R(95) Paracentral_Lobule_R(5)
49	-2.6	-48.5×15.3×33.0	Frontal_Inf_Oper_L	Frontal_Inf_Tri_L(47) Precentral_L(27) Frontal_Inf_Oper_L(26)
61	-2.6	38.5×-20.8×45.5	Postcentral_R	Postcentral_R(75) -(19) Precentral_R(6)
71	-2.6	-47.7×-45.1×-36.4	Cerebelum_Crus1_L	Cerebelum_Crus1_L(100)
186	-2.6	-26.4×-86.4×44.8		Occipital_Sup_L(40) -(34) Parietal_Sup_L(16) Parietal_Inf_L(10)
35	-2.6	-9.4×15.3×-12.0	Olfactory_L	Rectus_L(59) Olfactory_L(26) Caudate_L(15)
35	-2.6	-18.2×-27.4×-20.9	ParaHippocampal_L	ParaHippocampal_L(50) Cerebelum_4_5_L(45) Fusiform_L(5)
78	-2.7	0.2×3.5×5.7		-(100)
71	-2.7	-8.7×-75.4×47.7	Precuneus_L	Precuneus_L(91) Parietal_Sup_L(9)
101	-2.7	20.8×21.2×0.5	Putamen_R	Putamen_R(75) -(18) Caudate_R(7)
51	-2.8	20.8×30.1×35.9	Frontal_Sup_R	Frontal_Sup_R(87) Frontal_Mid_R(13)
454	-2.8	9.0×-14.9×6.4	Thalamus_R	Thalamus_R(92) -(8)
147	-2.8	42.2×-59.9×-54.1	Cerebelum_7b_R	Cerebelum_8_R(74) Cerebelum_7b_R(26)
105	-2.8	3.1×-6.0×41.8	Cingulum_Mid_R	Cingulum_Mid_R(100)
74	-2.9	-41.8×-72.4×27.1	Occipital_Mid_L	Occipital_Mid_L(93) Angular_L(7)
322	-2.9	-24.1×-57.7×69.1	Parietal_Sup_L	Parietal_Sup_L(100)
835	-3.0	-51.4×-0.1×35.9	Precentral_L	Precentral_L(74) Postcentral_L(21) Frontal_Inf_Oper_L(5)
69	-3.0	-0.5×-77.6×44.8	Precuneus_L	Precuneus_L(92) -(8)
788	-3.0	-35.9×-83.5×38.9	Occipital_Mid_L	Occipital_Mid_L(60) -(30) Parietal_Inf_L(10)
982	-3.0	-53.6×-56.9×50.7		Parietal_Inf_L(79) -(10) Angular_L(7) SupraMarginal_L(4)
705	-3.1	-44.8×-32.6×41.8	Parietal_Inf_L	Parietal_Inf_L(85) Postcentral_L(15)
973	-3.2	-41.8×-17.8×-30.5	Temporal_Inf_L	Temporal_Inf_L(54) -(42) Fusiform_L(4)
231	-3.2	-21.2×-36.3×-6.1	ParaHippocampal_L	ParaHippocampal_L(77) -(14) Lingual_L(6) Hippocampus_L(2) Precuneus_L(1)
1049	-3.3	54.0×-9.0×30.0	Postcentral_R	Postcentral_R(85) Precentral_R(11) -(3)
1510	-3.3	-27.1×-54.0×-6.1	Lingual_L	Fusiform_L(84) Lingual_L(14) Cerebelum_4_5_L(2)

1689	-3.3	54.0×-3.1×-36.4	Temporal_Inf_R	Temporal_Inf_R(94) -(5)
283	-3.3	14.9×-48.1×-45.2	Cerebelum_9_R	Cerebelum_9_R(99)
2347	-3.3	-8.7×-77.6×-45.2		-(68) Cerebelum_8_L(16) Cerebelum_7b_L(11) Cerebelum_Crus2_L(5)
2605	-3.3	-5.7×-62.8×57.3	Precuneus_L	Precuneus_R(46) Precuneus_L(38) Postcentral_R(10) -(4) Parietal_Sup_R(1)
3152	-3.4	-2.8×-20.8×41.8	Cingulum_Mid_L	Cingulum_Mid_L(48) Cingulum_Mid_R(31) Cingulum_Post_L(16) Precuneus_L(4) -(1)
2126	-3.5	-30.0×-56.9×44.8	Parietal_Inf_L	Parietal_Inf_L(48) Angular_L(24) Parietal_Sup_L(20) -(7) Occipital_Mid_L(2)
2616	-3.6	-24.1×-68.7×-32.7	Cerebelum_Crus1_L	Cerebelum_Crus1_L(92) Cerebelum_6_L(6) Cerebelum_Crus2_L(1) -(1)
209360	-5.1	-2.8×54.4×-12.0	Frontal_Mid_Orb_L	-(9) Frontal_Sup_Medial_L(4) Temporal_Mid_L(4) Frontal_Inf_Orb_R(4) Insula_R(4) Temporal_Mid_R(4) Frontal_Inf_Orb_L(4) Frontal_Mid_R(4) Temporal_Sup_R(3) Frontal_Mid_L(3) Cingulum_Ant_R(3) Frontal_Sup_Medial_R(3) Insula_L(3) Cingulum_Ant_L(3) Front

Table S4: Sensitivity analysis for predicting weight-change.

Abbreviations: SEN= Sensitivity, SPE= Specificity, BAC= Balanced accuracy, AUC= Area under the curve at 95% confidence interval.

Threshold	SEN (%)	SPE (%)	BAC (%)	AUC (%)
**T1**				
3%	20.7	77.8	49.2	0.49
5%	32.9	64.6	48.7	0.50
7%	66.0	35.3	50.6	0.48
**T2**				
3%	52.5	51.9	52.2	0.47
5%	37.0	53.4	45.2	0.40
7%	64.9	53.5	59.2	0.59

Table S5: Association between weight change and clinical outcomes. Pearson correlations (two-sided) were computed between weight change and symptom scale, separately at T1 and T2. Abbreviations: GAF = Global Assessment of Functioning, PANSS = Positive and Negative Syndrome Scale, BDI = Beck Depression Inventory, T1 = 1-year follow-up, T2 = 2-year follow-up, *r* = Pearson correlation, P = p-value, N = Sample size, ΔW1 = weight change at 1 year follow-up, ΔW1 = Weight change at 2 years follow-up

Clinical Measure	Timepoint	*r* (ΔW)	P (ΔW)	N (ΔW)
GAF Symptom	T1	-0.114	0.052	288
	T2	-0.229	0.002	174
GAF Disability	T1	-0.065	0.273	288
	T2	-0.144	0.058	174
PANSS Negative	T1	0.186	0.001	289
	T2	0.145	0.052	180
PANSS Positive	T1	0.086	0.145	289
	T2	0.067	0.372	180
PANSS General	T1	0.203	0.001	289
	T2	0.207	0.005	180
BDI	T1	0.071	0.291	221
	T2	0.021	0.812	129

Table S6: Association between weight change and medication dosage. Pearson correlations (two-sided) were computed between weight change and medication dosage, separately at T1 and T2. Abbreviations: *r* = Pearson correlation, P = p-value, N = Sample size, ΔW1 = weight change at 1-year follow-up, ΔW1 = Weight change at 2-years follow-up

Medication	Timepoint	*r*	P	N
All	T1	0.064624	0.37571	190
	T2	0.061083	0.52613	110
Weight gain	T1	0.21693	0.022821	110
	T2	0.18124	0.15862	62
Weight neutral	T1	-0.12649	0.40221	46
	T2	-0.26911	0.14322	31

Table S7: Correlation between BMIgap and weight change at 1-year follow-up.

The correlations across different age windows including 15–20, 20–25, 25–30, 30–35, and 35–40 years. Abbreviations: DW1= weight at T1 – Weight at T0, BMIgap= body mass index gap score, HC=Healthy controls, CHR= Clinical high-risk, ROD= Recent-onset depression, Net= any weight change without defining a threshold. Significant P values are highlighted in bold font.

	All			HC			CHR			ROD		
**ΔW1 (age bin (in yrs))**	***r***	**P**	**P_{FDR}**	***r***	**P**	**P_{FDR}**	***r***	**P**	**P_{FDR}**	***r***	**P**	**P_{FDR}**
**Net (15-20)**	0.1	0.27	0.707	0.15	0.26	0.99	-0.12	0.47	0.94	0.28	0.17	0.437
**3% (15-20)**	0.13	0.35	0.707	0.19	0.4	0.99	-0.19	0.41	0.94	0.41	0.15	0.437
**5% (15-20)**	0.14	0.39	0.707	0.21	0.45	0.99	-0.34	0.22	0.94	0.43	0.16	0.437
**7% (15-20)**	0.17	0.38	0.707	0.17	0.63	0.99	-0.36	0.25	0.94	0.8	0.03	0.437
**Net (20-25)**	0	0.98	0.98	0.03	0.72	0.99	0.01	0.91	0.97	0.01	0.93	0.95
**3% (20-25)**	-0.02	0.83	0.874	0.02	0.93	0.99	0.04	0.79	0.97	0.01	0.95	0.95
**5% (20-25)**	-0.04	0.72	0.847	-0.04	0.9	0.99	0.01	0.97	0.97	0.06	0.8	0.9
**7% (20-25)**	-0.1	0.53	0.707	-0.32	0.48	0.99	-0.07	0.76	0.97	0.22	0.47	0.769
**Net (25-30)**	0.03	0.72	0.847	0	0.98	0.99	0.03	0.88	0.97	0.08	0.63	0.81
**3% (25-30)**	0.12	0.35	0.707	0.08	0.71	0.99	0.3	0.41	0.94	0.12	0.58	0.803
**5% (25-30)**	0.13	0.46	0.707	-0.21	0.59	0.99	0.36	0.34	0.94	0.16	0.57	0.803
**7% (25-30)**	0.41	0.09	0.707	0.94	0.21	0.99	0.83	0.04	0.64	0.5	0.17	0.437
**Net (30-35)**	-0.03	0.79	0.874	0	0.99	0.99	-0.15	0.59	0.97	0.27	0.37	0.666
**3% (30-35)**	0.18	0.43	0.707	0.17	0.69	0.99	0.16	0.84	0.97	0.38	0.31	0.62
**5% (30-35)**	0.2	0.47	0.707	0.42	0.48	0.99	0.16	0.84	0.97	0.57	0.24	0.54
**7% (30-35)**	0.4	0.25	0.707	0.53	0.47	0.99	-	-	-	0.22	0.72	0.864
**Net (35-40)**	0.26	0.05	0.2	0.03	0.85	0.99	0.47	0.42	0.94	0.64	**0.01**	**0.04**
**3% (35-40)**	0.37	0.1	0.222	0.01	0.97	0.99	-1	-	-	0.95	9.8e-21	8.8e-13
**5% (35-40)**	0.72	0.03	0.2	0.1	0.87	0.99	-	-	-	1	7.2e-16	5.4e-10
**7% (35-40)**	0.85	0.07	0.2	-	-		-	-	-	1	4.3e-21	5.3e-10

Table S8: Correlation between BMIgap and weight change at 1-year follow-up.
The correlations across different age windows including 15-40, 20-40, 25-40, 30-40 years. Abbreviations: DW1= weight at T1 – Weight at T0, BMIgap= body mass index gap score, HC=Healthy controls, CHR= Clinical high-risk, ROD= Recent-onset depression, Net= any weight change without defining a threshold. Significant P values are highlighted in bold font.

	**All**			**HC**			**CHR**			**ROD**		
**ΔW1 (age bin (in yrs))**	***r***	**P**	**P_{FDR}**	***r***	**P**	**P_{FDR}**	***r***	**P**	**P_{FDR}**	***r***	**P**	**P_{FDR}**
**Net (15-40)**	0.05	0.22	0.314	0.07	0.23	0.99	-0.05	0.58	0.94	0.18	**0.04**	**0.053**
**3% (15-40)**	0.09	0.15	0.267	0.11	0.28	0.99	-0.01	0.94	0.94	0.21	0.06	0.075
**5% (15-40)**	0.1	0.2	0.308	0.1	0.48	0.99	-0.01	0.92	0.94	0.26	**0.05**	**0.051**
**7% (15-40)**	0.14	0.16	0.267	0.08	0.72	0.99	-0.1	0.54	0.94	0.45	**7.46e-16**	**2.73e-9**
**Net (20-40)**	0.03	0.54	0.54	0.02	0.8	0.99	-0.01	0.91	0.94	0.14	0.13	0.144
**3% (20-40)**	0.08	0.26	0.325	0.05	0.69	0.99	0.06	0.64	0.94	0.18	0.15	0.15
**5% (20-40)**	0.08	0.35	0.389	0.01	0.96	0.99	0.08	0.61	0.94	0.23	0.14	0.147
**7% (20-40)**	0.13	0.25	0.325	-0.02	0.94	0.99	-0.03	0.9	0.94	0.36	0.04	0.0727
**Net (25-40)**	0.06	0.37	0.389	0	0.99	0.99	-0.05	0.75	0.94	0.23	0.06	0.075
**3% (25-40)**	0.17	0.08	0.2	0.08	0.63	0.99	0.12	0.66	0.94	0.31	**0.05**	**0.055**
**5% (25-40)**	0.23	0.08	0.2	0.09	0.73	0.99	0.29	**0.03**	**0.047**	0.37	0.07	0.0824
**7% (25-40)**	0.46	0.01	0.2	0.24	0.57	0.99	0.6	**0.03**	**0.03**	0.53	**0.02**	**0.0467**
**Net (30-40)**	0.09	0.33	0.388	0.01	0.96	0.99	-0.16	0.49	0.94	0.41	**0.03**	**0.0487**
**3% (30-40)**	0.24	0.13	0.26	0.07	0.76	0.99	-0.09	0.87	0.94	0.57	0.03	0.0727
**5% (30-40)**	0.37	0.08	0.2	0.24	0.5	0.99	0.16	0.84	0.94	0.66	0.04	0.0727
**7% (30-40)**	0.5	0.06	0.2	0.36	0.56	0.99	-	-	-	0.64	0.06	0.075
**Net (35-40)**	0.26	0.05	0.2	0.03	0.85	0.99	0.47	0.42	0.94	0.64	**0.01**	**0.04**
**3% (35-40)**	0.37	0.1	0.222	0.01	0.97	0.99	-1	-	-	0.95	9.8e-21	8.8e-13
**5% (35-40)**	0.72	0.03	0.2	0.1	0.87	0.99	-	-	-	1	7.2e-16	5.4e-10
**7% (35-40)**	0.85	0.07	0.2	-	-		-	-	-	1	4.3e-21	5.3e-10

Table S9: Correlation between BMIgap and weight change at 2-year follow-up.
The correlations across different age windows including 15–20, 20–25, 25–30, 30–35, and 35–40 years. Abbreviations: DW2= weight at T2 – Weight at T0, BMIgap= body mass index gap score, HC=Healthy controls, CHR= Clinical high-risk, ROD= Recent-onset depression, Net= any weight change without defining a threshold. Significant P values are highlighted in bold font.

	All			HC			CHR			ROD		
**ΔW2 (age bin (in yrs))**	***r***	**P**	**P_{FDR}**	***r***	**P**	**P_{FDR}**	***r***	**P**	**P_{FDR}**	***r***	**P**	**P_{FDR}**
**Net (15-20)**	0.19	0.11	0.397	0.21	0.21	0.63	-0.13	0.58	0.98	0.52	**0.042**	**0.051**
**3% (15-20)**	0.27	0.13	0.397	0.17	0.5	0.682	0.25	0.56	0.98	0.76	**0.03**	**0.047**
**5% (15-20)**	0.27	0.19	0.397	0.21	0.5	0.682	-0.02	0.98	0.98	0.83	**0.02**	**0.049**
**7% (15-20)**	0.31	0.2	0.397	0.31	0.46	0.682	-0.02	0.98	0.98	0.83	**0.04**	**0.052**
**Net (20-25)**	0.02	0.81	0.81	-0.1	0.38	0.682	0.05	0.76	0.98	0.09	0.06	0.9
**3% (20-25)**	-0.07	0.6	0.671	-0.22	0.35	0.682	-0.03	0.86	0.98	-0.02	0.83	0.95
**5% (20-25)**	-0.17	0.26	0.412	-0.7	0.04	0.45	-0.05	0.81	0.98	-0.12	0.72	0.9
**7% (20-25)**	-0.24	0.21	0.397	-0.78	0.12	0.45	-0.19	0.47	0.98	0.11	0.8	0.9
**Net (25-30)**	0.16	0.13	0.397	0.22	0.11	0.45	0.05	0.84	0.98	0.13	0.58	0.9
**3% (25-30)**	0.21	0.23	0.397	0.26	0.34	0.682	0.21	0.62	0.98	0.19	0.56	0.9
**5% (25-30)**	0.2	0.39	0.494	0.27	0.6	0.75	0.26	0.57	0.98	0.16	0.7	0.9
**7% (25-30)**	0.43	0.17	0.397	-	-		0.27	0.66	0.98	0.65	0.23	0.591
**Net (30-35)**	0.05	0.78	0.81	0.39	0.07	0.45	0.51	0.24	0.98	-0.02	0.65	0.95
**3% (30-35)**	0.17	0.55	0.653	0.15	0.9	0.9	-	-	-	0.15	0.7	0.9
**5% (30-35)**	0.3	0.39	0.494	-	-	-	-	-	-	0.58	0.23	0.591
**7% (30-35)**	0.73	0.1	0.397	-	-	-	-	-	-	0.16	0.69	0.9
**Net (35-40)**	0.39	0.01	0.0347	0.06	0.77	0.77	0.56	0.44	0.87	0.73	**8.06e-9**	**0.00012**
**3% (35-40)**	0.63	0.02	0.0714	0.17	0.72	0.765	-	-	-	0.95	**0.01**	**0.05**
**5% (35-40)**	0.9	0.04	0.0514	-1	-	-	-	-	-	1	**0.03**	**0.0545**
**7% (35-40)**	1	0.03	0.0714	-	-	-	-	-	-	1	**0.03**	**0.0545**

Table S10: Correlation between BMIgap and weight change at 2-year follow-up.

The correlations across different age windows including 15-40, 20-40, 25-40, 30-40 years. Abbreviations: DW2= weight at T2 – Weight at T0, BMIgap= body mass index gap score, HC=Healthy controls, CHR= Clinical high-risk, ROD= Recent-onset depression, Net= any weight change without defining a threshold. Significant P values are highlighted in bold font.

	**All**			**HC**			**CHR**			**ROD**		
**ΔW2 (age bin (in yrs))**	***r***	**P**	**P_{FDR}**	***r***	**P**	**P_{FDR}**	***r***	**P**	**P_{FDR}**	***r***	**P**	**P_{FDR}**
**Net (15-40)**	0.14	**0.01**	**0.0347**	0.13	0.05	0.425	0.03	0.78	0.87	0.30	**0.01**	**0.05**
**3% (15-40)**	0.16	**0.04**	**0.0514**	0.12	0.37	0.765	0.09	0.54	0.87	0.32	**0.02**	**0.0545**
**5% (15-40)**	0.15	0.14	0.165	0.09	0.63	0.765	0.03	0.84	0.87	0.41	**0.02**	**0.0545**
**7% (15-40)**	0.18	0.13	0.162	0.11	0.69	0.765	-0.04	0.84	0.87	0.58	**7.06e-10**	**0.0001**
**Net (20-40)**	0.12	0.04	0.0714	0.11	0.16	0.68	0.08	0.51	0.87	0.22	**0.05**	**0.0714**
**3% (20-40)**	0.12	0.19	0.211	0.08	0.62	0.765	0.04	0.79	0.87	0.23	0.15	0.158
**5% (20-40)**	0.08	0.49	0.49	-0.11	0.65	0.765	0.03	0.87	0.87	0.28	0.16	0.16
**7% (20-40)**	0.1	0.49	0.49	-0.31	0.46	0.765	-0.09	0.700	0.87	0.47	**0.03**	**0.0545**
**Net (25-40)**	0.18	**0.02**	**0.0414**	0.23	0.02	0.34	0.12	**0.027**	**0.049**	0.28	**0.05**	0.0714
**3% (25-40)**	0.27	**0.03**	**0.0547**	0.23	0.26	0.765	0.28	**0.049**	**0.049**	0.34	0.09	0.1
**5% (25-40)**	0.33	0.05	0.0714	0.33	0.36	0.765	0.36	**0.031**	**0.049**	0.43	0.09	0.1
**7% (25-40)**	0.46	**0.01**	**0.0347**	0.76	0.45	0.765	0.27	**0.002**	**0.01**	0.63	**0.02**	**0.0545**
**Net (30-40)**	0.21	0.05	0.0714	0.22	0.15	0.68	0.52	0.09	0.87	0.43	**0.03**	**0.0545**
**3% (30-40)**	0.35	0.08	0.107	0.23	0.53	0.765	0.96	0.19	0.87	0.48	0.08	0.1
**5% (30-40)**	0.52	0.05	0.0716	0.38	0.62	0.765	1	-	-	0.68	**0.04**	**0.0547**
**7% (30-40)**	0.71	0.03	**0.0547**	-	-	-	-	-	-	0.67	0.07	0.0933
**Net (35-40)**	0.39	0.01	0.0347	0.06	0.77	0.77	0.56	0.44	0.87	0.73	**8.06e-9**	**0.00012**
**3% (35-40)**	0.63	0.02	0.0714	0.17	0.72	0.765	-	-	-	0.95	**0.01**	**0.05**
**5% (35-40)**	0.9	0.04	0.0514	-1	-	-	-	-	-	1	**0.03**	**0.0545**
**7% (35-40)**	1	0.03	0.0714	-	-	-	-	-	-	1	**0.03**	**0.0545**

References

1. Wolfers T, Doan NT, Kaufmann T, Alnæs D, Moberget T, Agartz I, *et al.* (2018): Mapping the Heterogeneous Phenotype of Schizophrenia and Bipolar Disorder Using Normative Models. *JAMA Psychiatry* 75: 1146–1155.
2. Koutsouleris N, Meisenzahl EM, Borgwardt S, Riecher-Rössler A, Frodl T, Kambeitz J, *et al.* (2015): Individualized differential diagnosis of schizophrenia and mood disorders using neuroanatomical biomarkers. *Brain J Neurol* 138: 2059–2073.
3. Koutsouleris N, Kambeitz-Ilankovic L, Ruhrmann S, Rosen M, Ruef A, Dwyer DB, *et al.* (2018): Prediction Models of Functional Outcomes for Individuals in the Clinical High-Risk State for Psychosis or With Recent-Onset Depression: A Multimodal, Multisite Machine Learning Analysis. *JAMA Psychiatry* 75: 1156–1172.
4. Correll CU, Lencz T, Malhotra AK (2011): Antipsychotic drugs and obesity. *Trends Mol Med* 17: 97–107.
5. Franke K, Gaser C (2019): Ten Years of BrainAGE as a Neuroimaging Biomarker of Brain Aging: What Insights Have We Gained? *Front Neurol* 10. https://doi.org/10.3389/fneur.2019.00789
6. Franke K, Ziegler G, Klöppel S, Gaser C (2010): Estimating the age of healthy subjects from T1-weighted MRI scans using kernel methods: Exploring the influence of various parameters. *NeuroImage* 50: 883–892.
7. Rutherford S, Kia SM, Wolfers T, Fraza C, Zabihi M, Dinga R, *et al.* (2022): The normative modeling framework for computational psychiatry. *Nat Protoc* 17: 1711–1734.
8. Marquand AF, Rezek I, Buitelaar J, Beckmann CF (2016): Understanding Heterogeneity in Clinical Cohorts Using Normative Models: Beyond Case-Control Studies. *Biol Psychiatry* 80: 552–561.
9. Kia SM, Huijsdens H, Dinga R, Wolfers T, Mennes M, Andreassen OA, *et al.* (2020): Hierarchical Bayesian Regression for Multi-site Normative Modeling of Neuroimaging Data. In: Martel AL, Abolmaesumi P, Stoyanov D, Mateus D, Zuluaga MA, Zhou SK, *et al.*, editors. *Medical Image Computing and Computer Assisted Intervention – MICCAI 2020*. Cham: Springer International Publishing, pp 699–709.
10. Dwyer DB, Falkai P, Koutsouleris N (2018): Machine Learning Approaches for Clinical Psychology and Psychiatry. *Annu Rev Clin Psychol* 14: 91–118.
11. Wolfers T, Buitelaar JK, Beckmann CF, Franke B, Marquand AF (2015): From estimating activation locality to predicting disorder: A review of pattern recognition for neuroimaging-based psychiatric diagnostics. *Neurosci Biobehav Rev* 57: 328–349.
12. Gómez-Verdejo V, Parrado-Hernández E, Tohka J, Alzheimer's Disease Neuroimaging Initiative (2019): Sign-Consistency Based Variable Importance for Machine Learning in Brain Imaging. *Neuroinformatics* 17: 593–609.
13. Rolls ET, Huang C-C, Lin C-P, Feng J, Joliot M (2020): Automated anatomical labelling atlas 3. *NeuroImage* 206: 116189.
14. Bayer J, Thompson P, Ching C, Liu M, Chen A, Panzenhagen A, *et al.* (2022): Site effects how-to and when: An overview of retrospective techniques to accommodate site effects in multi-site neuroimaging analyses. *Front Neurol* 13: 923988.
15. Jovicich J, Czanner S, Greve D, Haley E, van der Kouwe A, Gollub R, *et al.* (2006): Reliability in multi-site structural MRI studies: effects of gradient non-linearity correction on phantom and human data. *NeuroImage* 30: 436–443.
16. Adin A, Krainski ET, Lenzi A, Liu Z, Martínez-Minaya J, Rue H (2024): Automatic cross-validation in structured models: Is it time to leave out leave-one-out? *Spat Stat* 62: 100843.

17. Koutsouleris N, Dwyer DB, Degenhardt F, Maj C, Urquijo-Castro MF, Sanfelici R, *et al.* (2021): Multimodal Machine Learning Workflows for Prediction of Psychosis in Patients With Clinical High-Risk Syndromes and Recent-Onset Depression. *JAMA Psychiatry* 78: 195–209.
18. Krishnan A, Williams LJ, McIntosh AR, Abdi H (2011): Partial Least Squares (PLS) methods for neuroimaging: a tutorial and review. *NeuroImage* 56: 455–475.
19. McIntosh AR, Lobaugh NJ (2004): Partial least squares analysis of neuroimaging data: applications and advances. *NeuroImage* 23 Suppl 1: S250-263.
20. Popovic D, Ruef A, Dwyer DB, Antonucci LA, Eder J, Sanfelici R, *et al.* (2020): Traces of Trauma: A Multivariate Pattern Analysis of Childhood Trauma, Brain Structure, and Clinical Phenotypes. *Biol Psychiatry* 88: 829–842.
21. Thomas Yeo BT, Krienen FM, Sepulcre J, Sabuncu MR, Lashkari D, Hollinshead M, *et al.* (2011): The organization of the human cerebral cortex estimated by intrinsic functional connectivity. *J Neurophysiol* 106: 1125–1165.
22. Baker JT, Holmes AJ, Masters GA, Yeo BTT, Krienen F, Buckner RL, Öngür D (2014): Disruption of cortical association networks in schizophrenia and psychotic bipolar disorder. *JAMA Psychiatry* 71: 109–118.
23. Efron B, Tibshirani R (1986): Bootstrap Methods for Standard Errors, Confidence Intervals, and Other Measures of Statistical Accuracy. *Stat Sci* 1: 54–75.
