# Supplementary material for: The BMIgap tool to quantify transdiagnostic brain signatures of current and future weight
Source: Nat Ment Health. 2025 Oct 20;3(11):1395–406. doi: 10.1038/s44220-025-00522-3 (PMC12589130; doi:10.1038/s44220-025-00522-3)
Supplement: Supplementary file 2 — Reporting Summary [file 44220_2025_522_MOESM2_ESM.pdf]

nature portfolio

Corresponding author(s): Nikolaos Koutsouleris

Last updated by author(s): Aug 21, 2025

Reporting Summary

Nature Portfolio wishes to improve the reproducibility of the work that we publish. This form provides structure for consistency and transparency in reporting. For further information on Nature Portfolio policies, see our Editorial Policies and the Editorial Policy Checklist.

Statistics

For all statistical analyses, confirm that the following items are present in the figure legend, table legend, main text, or Methods section.

n/a	Confirmed	
☐	☒	The exact sample size (*n*) for each experimental group/condition, given as a discrete number and unit of measurement
☐	☒	A statement on whether measurements were taken from distinct samples or whether the same sample was measured repeatedly
☐	☒	The statistical test(s) used AND whether they are one- or two-sided<br>*Only common tests should be described solely by name; describe more complex techniques in the Methods section.*
☐	☒	A description of all covariates tested
☐	☒	A description of any assumptions or corrections, such as tests of normality and adjustment for multiple comparisons
☐	☒	A full description of the statistical parameters including central tendency (e.g. means) or other basic estimates (e.g. regression coefficient) AND variation (e.g. standard deviation) or associated estimates of uncertainty (e.g. confidence intervals)
☐	☒	For null hypothesis testing, the test statistic (e.g. *F*, *t*, *r*) with confidence intervals, effect sizes, degrees of freedom and *P* value noted<br>*Give P values as exact values whenever suitable.*
☒	☐	For Bayesian analysis, information on the choice of priors and Markov chain Monte Carlo settings
☐	☒	For hierarchical and complex designs, identification of the appropriate level for tests and full reporting of outcomes
☐	☒	Estimates of effect sizes (e.g. Cohen's *d*, Pearson's *r*), indicating how they were calculated

Our web collection on statistics for biologists contains articles on many of the points above.

Software and code

Policy information about availability of computer code

Data collection	No data have been explicitly collected for this specific study.; only pre-existing datasets were used. MRI image processing was conducted using VBM8, with detailed settings provided in the Supplementary Material. The exact SPM batch file with all parameters is available upon request from the corresponding author. All other analysis code is available via the GitHub repository.
Data analysis	NeuroMiner 1.2 (https://neurominer-git.github.io/NeuroMiner_1.2/intro.html), SPLS Toolbox (https://github.com/dpopovic30/spls_toolbox_compiled), MATLAB R2022a, MATLAB R2023b, R version 4.4.1, RStudio Version 2024.12.1+563, and VBM8 were used in the analyses. The NeuroMiner template files detailing the machine learning analysis setup are described in the manuscript and the exact template files may be available upon request from the corresponding author.

For manuscripts utilizing custom algorithms or software that are central to the research but not yet described in published literature, software must be made available to editors and reviewers. We strongly encourage code deposition in a community repository (e.g. GitHub). See the Nature Portfolio guidelines for submitting code & software for further information.

Data

Policy information about availability of data

All manuscripts must include a data availability statement. This statement should provide the following information, where applicable:

- Accession codes, unique identifiers, or web links for publicly available datasets
- A description of any restrictions on data availability
- For clinical datasets or third party data, please ensure that the statement adheres to our policy

Parts of the data, including the IXI dataset (https://brain-development.org/ixi-dataset/) and the Cambridge Centre for Ageing and Neuroscience (CAM-CAN) dataset (https://camcan-archive.mrc-cbu.cam.ac.uk), are accessible to researchers upon request from the respective repositories. Other datasets (PRONIA, MUC, NORMENT) analyzed during the current study are not publicly available due to data sharing restrictions defined in the participants' signed informed consent agreements. Trained models are available from the corresponding author upon reasonable request.
The code supporing the findings of this study is available via GitHub at https://github.com/adyasha95/BMIgapCodeRepo.

Research involving human participants, their data, or biological material

Policy information about studies with human participants or human data. See also policy information about sex, gender (identity/presentation), and sexual orientation and race, ethnicity and racism.

Reporting on sex and gender	The term sex is used in the manuscript to describe biological attributes. Both males and females were included in the study, with group-specific numbers reported. Sex-specific analyses were performed, with male and female groups separately assessed for the association between weight change and BMIgap.
Reporting on race, ethnicity, or other socially relevant groupings	Race and ethnicity data were not consistently collected across all cohorts and were therefore not included in the analysis.
Population characteristics	The study included 1504 healthy controls (HC; discovery: N=770, mean age=41.3±15.5 years, 56.5% female; validation: N=734, mean age=32.2±12.8 years, 50.8% female; Cam-CAN: N=536, mean age=54.3±18.6 years, 48.7% female), 146 individuals with schizophrenia (N=146, mean age=30.8±10.0 years, 23.3% female), 213 with clinical high-risk states (CHR; N=213, mean age=23.9±5.2 years, 48.4% female), and 200 with recent-onset depression (ROD; N=200, mean age=26.0±6.4 years, 48.0% female).
Recruitment	Participants included are from independent datasets covering 15 sites: Information eXtraction from Images (IXI; https://brain-development.org/ixi-dataset/), Personalized Prognostic Tools for Early Psychosis Management (PRONIA; www.pronia.eu), Norwegian Centre for Mental Disorders Research (https://www.med.uio.no/norment/), the Munich Brain Imaging Database and Cambridge Centre for Ageing and Neuroscience dataset. Patients with schizophrenia were assessed using the Structured Clinical Interview for DSM-IV Axis I Disorders (SCID). Recent-onset depression (ROD) was defined as a first major depressive episode within the past three months, determined by SCID. Clinical high-risk (CHR) states were defined by cognitive disturbances assessed with the Schizophrenia Proneness Instrument and/or ultra-high-risk criteria based on the Structured Interview for Psychosis-Risk Syndromes. For CHR and ROD groups, only minimal antipsychotic medication use was permitted.
Ethics oversight	Ethical approval was obtained from the institutional review boards or ethics committees at each participating site. All participants provided written informed consent in accordance with the Declaration of Helsinki.

Note that full information on the approval of the study protocol must also be provided in the manuscript.

Field-specific reporting

Please select the one below that is the best fit for your research. If you are not sure, read the appropriate sections before making your selection.

☒ Life sciences ☐ Behavioural & social sciences ☐ Ecological, evolutionary & environmental sciences

For a reference copy of the document with all sections, see nature.com/documents/nr-reporting-summary-flat.pdf

Life sciences study design

All studies must disclose on these points even when the disclosure is negative.

Sample size	Sample size was determined based on availability of suitable data across multiple independent cohorts. While no formal a priori power calculation was performed, the inclusion of 770 healthy controls for model training, 734 for validation, 536 for external validation, and 559 clinical participants (146 SCZ,213 CHR, 200 ROD) provided sufficient power for predictive modeling and subgroup comparisons. Reporting follows the TRIPOD guidelines.
Data exclusions	Participants were excluded if T1-weighted MRI data were missing, of insufficient quality (e.g., motion artifacts), or failed preprocessing steps such as segmentation or normalization in the VBM8 pipeline. Exclusion criteria were pre-specified and consistently applied across all sites.
Replication	The model was validated in two independent healthy control cohorts (HCvalidation and HCCam-CAN) to test generalizability and

Replication	reproducibility of the findings. Results were further applied to three clinical samples (SCZ, CHR, ROD), with consistent patterns observed across subgroups. All model evaluation steps were conducted within a strict nested cross-validation framework to ensure generalizability.
Randomization	Randomization was not applicable, as this was an observational, non-interventional study using previously collected clinical and neuroimaging data. No random group allocation or experimental manipulation was involved.
Blinding	Blinding was not applicable in this observational study. However, data preprocessing and model training were fully automated and performed using nested cross-validation to prevent information leakage between training and test data.

Reporting for specific materials, systems and methods

We require information from authors about some types of materials, experimental systems and methods used in many studies. Here, indicate whether each material, system or method listed is relevant to your study. If you are not sure if a list item applies to your research, read the appropriate section before selecting a response.

Materials & experimental systems

n/a	Involved in the study	
☒	☐	Antibodies
☒	☐	Eukaryotic cell lines
☒	☐	Palaeontology and archaeology
☒	☐	Animals and other organisms
☐	☒	Clinical data
☒	☐	Dual use research of concern
☒	☐	Plants

Methods

n/a	Involved in the study	
☒	☐	ChIP-seq
☒	☐	Flow cytometry
☐	☒	MRI-based neuroimaging

Clinical data

Policy information about clinical studies

All manuscripts should comply with the ICMJE guidelines for publication of clinical research and a completed CONSORT checklist must be included with all submissions.

Clinical trial registration	The study is not part of any clinical trials
Study protocol	This study combined data from multiple sources. Data from the PRONIA consortium (www.pronia.eu) were collected following a standardized, multicenter recruitment and assessment protocol across sites in Finland, Germany, Italy, Switzerland, and the United Kingdom. Data from the Cam-CAN project (www.cam-can.org) and the IXI dataset (https://brain-development.org/ixi-dataset/) are publicly available and were collected according to their respective study protocols, available on their websites.<br>Data from the NORMENT Centre for Mental Disorders Research (www.med.uio.no/norment/english/) were collected under local ethics approvals at the University of Oslo and Oslo University Hospital, following standardized clinical and MRI acquisition protocols. Data from the Munich Brain Imaging Database were collected under institutional guidelines and ethical approvals at LMU Munich. Standardized MRI and clinical assessment protocols were followed across sites where applicable. All participants provided written informed consent in accordance with the Declaration of Helsinki.
Data collection	No data have been collected during this specific study. We have used only existing and existing datasets in this study. For PRONIA, participants underwent baseline and follow-up assessments every three months. Public datasets (Cam-CAN and IXI) provide cross-sectional demographic, cognitive, and MRI data collected at their respective sites. NORMENT and MUC datasets included clinical participants and healthy controls recruited through clinical services and community outreach, with corresponding MRI and clinical evaluations. Patients with schizophrenia were assessed using the Structured Clinical Interview for DSM-IV Axis I Disorders (SCID). Recent-onset depression (ROD) was defined as a first major depressive episode within the past three months, determined by SCID. Clinical high-risk (CHR) states were defined by cognitive disturbances assessed with the Schizophrenia Proneness Instrument and/or ultra-high-risk criteria based on the Structured Interview for Psychosis-Risk Syndromes. For CHR and ROD groups, only minimal antipsychotic medication use was permitted.
Outcomes	The primary outcome was the brain-based deviation from expected BMI (BMIgap), derived from structural MRI data to capture neurobiological markers of metabolic vulnerability. Secondary outcomes included clinical symptom scores, functional outcomes, and future weight change trajectories, depending on cohort data availability.

Plants

Seed stocks	NA
Novel plant genotypes	NA
Authentication	NA

Magnetic resonance imaging

Experimental design

Design type	Structural MRI, resting state (no task)
Design specifications	Structural T1-weighted images were acquired across multiple sites using standardized protocols. No experimental trials or tasks were administered; the MRI sessions consisted solely of anatomical scans.
Behavioral performance measures	NA

Acquisition

Imaging type(s)	Structural (T1-weighted MRI)
Field strength	1.5 Tesla and 3 Tesla scanners were used across different sites.
Sequence & imaging parameters	High-resolution T1-weighted anatomical scans were obtained using different MRI scanners (Siemens, Philips, GE). Voxel sizes ranged from 0.45×0.45×1.5 mm^3 to 1×1×1 mm^3. Field of View (FOV) ranged approximately between 230×230 mm^2 and 288×288 mm^2.<br>Typical TR: 2000–2730 ms;<br>TE: 2.28–5.5 ms;<br>Flip Angle: 7°–12°.<br>Pulse sequences varied slightly across scanners but were all optimized for structural brain imaging.<br>(details in the Supplement Methods)
Area of acquisition	Whole-brain anatomical scans covering the entire brain volume were acquired.

Diffusion MRI ☐ Used ☒ Not used

Preprocessing

Preprocessing software	VBM8 toolbox (http://dbm.neuro.uni-jena.de/vbm) implemented in SPM.<br>Steps included: bias correction, tissue classification, normalization to MNI-space, modulation by non-linear components only, and reslicing to 3×3×3 mm^3 isotropic voxel size for computational efficiency and noise reduction.
Normalization	Images were normalized to MNI space using a unified model with both linear (12-parameter affine) and non-linear transformations, including high-dimensional DARTEL normalization.
Normalization template	MNI standard space template (as implemented in VBM8).
Noise and artifact removal	Bias correction was applied to correct for intensity inhomogeneities.<br>An absolute masking threshold of 0.1 was used to exclude voxels of non-grey matter tissues. No explicit motion correction was necessary as only structural MRI data were analyzed.
Volume censoring	NA

Statistical modeling & inference

Model type and settings	Multivariate predictive modeling using machine learning (support vector regression) within a normative modeling framework. Five-fold repeated nested cross-validation with five permutations at inner and outer loops to prevent overfitting and information leakage.

Effect(s) tested

Individualized prediction of BMI from voxel-wise GMV patterns across healthy and clinical populations; deviation scores (BMIgap) were analyzed across diagnostic groups.

Specify type of analysis: ☒ Whole brain ☐ ROI-based ☐ Both

Statistic type for inference

(See Eklund et al. 2016)

Voxel-wise analysis.

Correction

False Discovery Rate (FDR) correction for multiple comparisons.

Models & analysis

n/a	Involved in the study
☒	☐ Functional and/or effective connectivity
☒	☐ Graph analysis
☐	☒ Multivariate modeling or predictive analysis

Multivariate modeling and predictive analysis

Vol-wise grey matter volume (GMV) was used as the independent variable (71276 features per subject).
Feature extraction and dimensionality reduction were performed using principal component analysis (PCA) at different energy thresholds (0.25, 0.50, 0.75) during cross-validation.
Model training was performed using ν-support vector regression (ν-SVR) with a linear kernel.
A repeated nested five-fold cross-validation with five permutations was used to prevent overfitting and ensure model generalizability. Model evaluation was based on the mean absolute error (MAE) as the performance metric. Statistical significance of the model performance was assessed using 1000 label permutations ($\alpha = 0.05$).
The final predictive brain patterns were visualized based on cross-validation ratio and sign-consistency mapping.
